# Sustainable xanthophylls-containing poly(ε-caprolactone)s: synthesis, characterization, and use in green lubricants†

**DOI:** 10.1039/d2ra04502h

**Published:** 2022-10-27

**Authors:** Eloy Rodríguez-deLeón, Moustapha Bah, José E. Báez, María T. Hernández-Sierra, Karla J. Moreno, Alejandro Nuñez-Vilchis, José Bonilla-Cruz, Kenneth J. Shea

**Affiliations:** Posgrado en Ciencias Químico Biológicas, Faculty of Chemistry, Autonomous University of Queretaro (UAQ) Cerro de Las Campanas Querétaro 76010 Mexico eloy.rodriguez@uaq.mx; Department of Chemistry, Division of Natural and Exact Sciences, University of Guanajuato (UG), Campus Guanajuato Noria Alta S/N Guanajuato 36050 Mexico jebaez@ugto.mx; Department of Mechanical Engineering, National Technology Institute of Mexico at Celaya Celaya 38010 Guanajuato Mexico; Centro de Investigación en Materiales Avanzados S.C. (CIMAV-Monterrey) Av. Alianza Norte 202, PIIT, Autopista Monterrey-Aeropuerto Km 10 Apodaca 66628 N.L. Mexico; Deparment of Chemistry, University of California, Irvine, (UCI) Irvine 92697-2025 California USA

## Abstract

Three xanthophylls [(3*R*,3′*R*,6′*R*)-lutein (1), (3*R*,3′*S*)-zeaxanthin (2), and (3*R*,3′*S*)-astaxanthin (3)] were used for the first time as initiators in the ring-opening polymerization (ROP) of ε-caprolactone (CL) catalyzed by tin(ii) 2-ethylhexanoate [Sn(Oct)_2_] for the synthesis of novel sustainable xanthophyll-containing poly(ε-caprolactone)s (xanthophylls-PCL). The obtained polyesters were characterized by ^1^H and ^13^C NMR, FT-IR, DSC, SEC, and MALDI-TOF MS, and their use as additives in green lubricants was evaluated using a sliding friction test under boundary conditions. Xanthophylls-PCL were obtained with good conversions and with molecular weights determined by SEC to be between 2500 and 10 500 Da. The thermal properties of xanthophyll-polyesters showed a crystalline domain, detected by DSC. Lastly, the green lubricant activity of these polymers was evaluated and the results showed that xanthophylls-PCL could be employed as additives for biodegradable lubricant applications since they have better tribological behavior than current additives, which demonstrates their potential as future commercial materials with interesting eco-friendly properties for diverse applications.

## Abstract 2

Sustainable polyesters initiators from renewable resources and additives in green lubricants.

## Graphical abstract

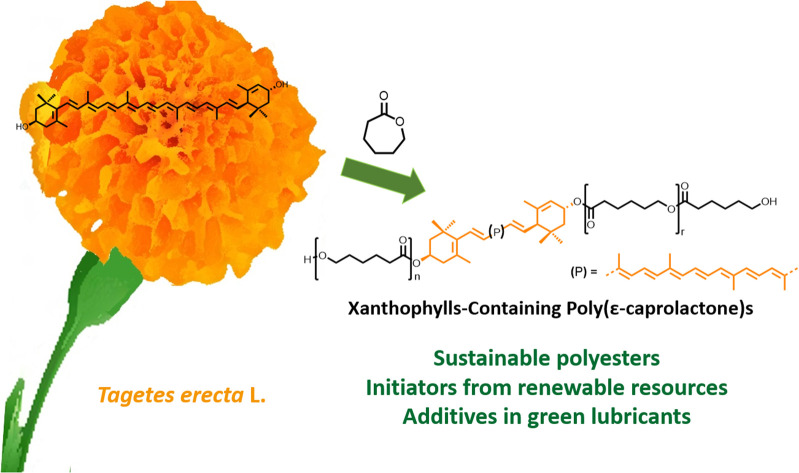

## Introduction

**Fig. 1:**
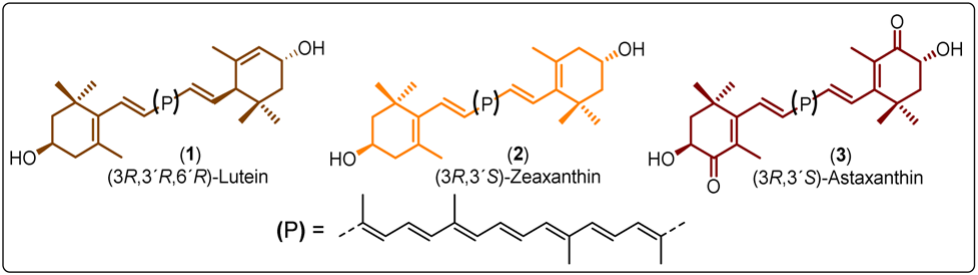
Structure of xanthophylls used in this work.

**Scheme 1:**
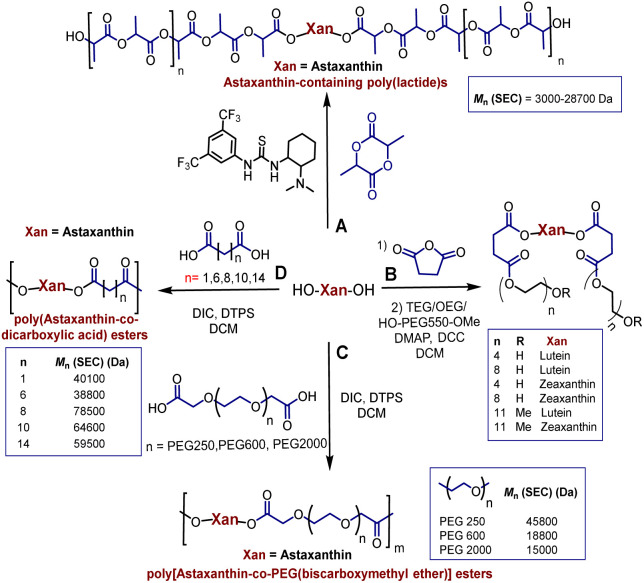
Synthesis of polymeric esters using xanthophylls.

Polyesters derived from renewable resources are nowadays an expanding area with growing scientific activity,^1–3^ due to the growing need for polymers that are currently obtained from petroleum-based resources.^4–6^ Moreover, sustainability is a relevant topic today and for future generations.^7^ In this sense, at a global level, petroleum as a starting material for plastics production has limited availability.^4^ For this reason, natural products represent an alternative to produce biodegradable and eco-friendly materials.^8,9^ Among all the widespread compounds present in nature, carotenoids stand out as they perform important and crucial roles in the organisms in which they are present. For instance, carotenoids are responsible for yellow, orange, and red colors of birds'plumage and scales of some fish.^10,11^ In these animals, the intensity and brightness of their colors seem to be an indicator of good health.^12^ Also, carotenoids are selectively accumulated in the human macula lutea, in the center of the retina, where they comply with eye protection functions.^13,14^ Different studies have corroborated that xanthophylls such as lutein (1) and zeaxanthin (2) are efficient to prevent age-related macular degeneration (AMD).^15–17^ Carotenoids are a family of compounds that are composed of two classes: carotenes and xanthophylls. Chemically, carotenes are those formed only by carbon and hydrogen. Examples of these are α- and β-carotenes, and lycopene. Xanthophylls, on the other hand, are oxygenated carotenes with epoxy, hydroxy, methoxy or carbonyl groups. Examples of these are: lutein (1), zeaxanthin (2), and astaxanthin (3) (Fig. 1).^18^ One important function of the carotenoids in organisms is their protective action against reactive oxygen species (ROS).^10,19^ This feature can take advantage to create new materials that own the intrinsic characteristics of xanthophylls such as their antioxidant properties. Previously, our group reported the use of compounds 1, 2, and 3 as chain extender agents in poly(ester-urethane)s synthesis.^20^ Likewise, there are various studies in which carotenoids are used as functional diols to obtain diverse polymeric esters (Scheme 1).

As illustred in Scheme 1, astaxanthin was used as initiator in the ring opening polymerization (ROP) of d,l-lactide in presence of a bifunctional organocatalyst (thiourea/tertiary amine) to obtain astaxanthin-containing poly(lactide)s (route A).^21^ Route B shows the procedure leading to the synthesis of poly(ethylene glycols) (PEGs) from lutein or zeaxanthin disuccinate conjugated with tetra, octa or poly(ethylene glycol).^22^ Route C represents a copolymerization of astaxanthin with various polyethyleneglycol-[biscarboxymethyl ether]s, while in route D, compound 3 was used with different diacids resulting in “polyactive” polyesters (antimicrobial agents against *Staphylococcus aureus* MRSA252 and MSSA476).^23–25^ It is worth highlighting that the stereochemistry of the astaxanthin used in routes A, C and D was not defined, while the stereochemistries of lutein and zeaxanthin used in route B were (3*R*,3′*R*,6′*R*) and (3*R*,3′*R*), respectively.

Xanthophylls such as 1, 2, and 3 have never been used before as initiators to produce poly(ε-caprolactone)s. In the present contribution, we synthesized novel xanthophylls-containing poly(ε-caprolactone)s with important characteristics due to the fact that they are biocompatible and biodegradable materials,^26–29^ and can be used as additives in green lubrication.

## Experimental section

### Materials

(3*R*,3′*R*,6′*R*)-lutein (1) was extracted from marigold oleoresin, which was purchased from Alcosa Biotech (Alcosa Biotech, Apaseo el Grande, Guanajuato, México). (3*R*,3′*S*)-zeaxanthin (2) and (3*R*,3′*S*)-astaxanthin (3) were obtained by partial synthesis from compound 1 (as we have described previously^30^). ε-caprolactone, 1,8-octanediol, tin(ii) 2-ethylhexanoate, and deuterated chloroform (CDCl_3_) were acquired from Sigma Aldrich Co (St Louis, Mo, USA) and used as received.

### Instruments

Fourier transform-infrared (FTIR) spectra of all polyesters and xanthophylls were obtained with the attenuated total reflectance spectroscopy (ATR) technique in a PerkinElmer Spectrum 100 spectrometer. Nuclear magnetic resonance (NMR) spectra of ^1^H and ^13^C were recorded on a 500 MHz Bruker Avance III HD instrument and CDCl_3_ as solvent. Spectra were referenced to the chemical shift of the residual solvent protons at 7.26 ppm in the ^1^H NMR and the residual solvent carbons at 77.16 ppm in the ^13^C NMR spectrum.

Size exclusion chromatograph 1260 Infinity Agilent Technologies coupled to a refractive index detector (1260 RID) was used to determine the number average molecular weight (*M*_n_) and dispersity (*Đ*_M_) of the synthesized polyesters. These analyses were recorded using as stationary phase a column PLgel 5 mm MIXED-D and THF as mobile phase, at a flow rate of 1.0 mL min^−1^. All chromatograms were acquired at 37 °C and the results reported relative to polystyrene standards.

Differential Scanning Calorimetry (DSC) thermograms were performed on a TA Q200 instrument. Samples were sealed in aluminum pans. The thermal analysis was obtained using the next method: three scans were performed with two heating cycles (from 0 to 100 °C, 100 to −30 °C, and −30 to 100 °C). The flow rate of cooling/heating was 10 °C min^−1^ under nitrogen purge. All data presented are taken from the second heating cycle and the melting points (*T*_m_) are given as the minimum of the endothermic transition.

Matrix-assisted laser desorption ionization time-of-flight (MALDI-TOF) spectra were recorded in linear mode by using an AB SCIEX TOF/TOF 5800 SYSTEM equipped with a nitrogen laser emitting at 349 nm, an input bandwith = 1000 MHz with a 3 ns pulse width and working in positive mode. 2,5-Dihydroxybenzoyc acid was used as matrix in a concentration of 10 mg mL^−1^ in THF as solvent. Polyester samples were dissolved in THF (3 mg mL^−1^), then 10 μL of sample solution was mixed with 10 μL of matrix solution. Different aliquots were placed on a stainless-steel plate and the solvent was evaporated before starting the acquisition.

### Synthesis of xanthophylls-containing poly(ε-caprolactone)s (HOPCLOH)

Polymerization reaction was performed in bulk into a dry vial, in which ε-caprolactone (50 mmol, 5.707 g), tin(ii) 2-ethylhexanoate (two drops, 24 mg), and 2.5 mmol of the corresponding xanthophyll (compound 1, 2, or 3) were placed. The mixture was stirred and heated at 120 °C for different periods of time (20 hours to 48 hours). The polyesters obtained were analyzed and used without purification. Three different macrodiols derived from each xanthophyll and CL were obtained by ROP. ^1^H NMR data of the polyester derived from zeaxanthin (zeaxanthin-PCL) (Fig. S6,† 500 MHz, CDCl_3_, ppm): *δ* 6.47 [m, 4H, (11,11′ & 15,15′, –CH̲=CH–), zeaxanthin], 6.20 [m, 2H, (12,12′, –CH̲=CH–), zeaxanthin], 6.17 [m, 4H, (14,14′ & 10,10′, –CH̲=CH–), zeaxanthin], 6.09 [m, 2H, (8,8′, –CH̲=CH–), zeaxanthin], 5.97 [m, 2H, (7,7′, –CH̲=CH–), zeaxanthin], 5.00 [m, 2H, (3,3′, –CH̲–O–), zeaxanthin], 4.08 [t, 2H, (f, –CH_2_–O–), PCL], 3.58 [t, 2H, (f′, –CH_2_–OH), PCL], 2.49 [t, 2H, (b, –CH_2_–CO–), PCL], 1.90 [s, 12H, (19,19′ & 20,20′, –CH_3_), zeaxanthin], 1.62 [m, 4H, (c & e, –CH_2_–), PCL], 1.10 [m, 2H, (d, –CH_2_–), PCL], 0.92 [s, 6H, (16,17, –CH_3_), zeaxanthin]. M_*n*_(SEC) = 8023, *Đ*_M_ = 1.48. FT-IR (cm^−1^; Fig. S15†): 3438 (*v* O–H), 2944 (*v*_as_ CH_2_), 2865 (*v*_s_ CH_2_), 1721 (*v* C=O), 1176 [*v*_as_ C–(C=O)–O], 1045 (*v*, C–O), 960 (*ω*, C=C–H), 731 (*ρ*, CH_2_). DSC data (Table 2): 51.7 and 53.5 °C, Δ*H*_m_ = 77.97 J g^−1^, *χ*_i_ = 58%.

### Evaluation of xanthophylls-PCL as additives for biodegradable lubricants

The polyesters derived from lutein, zeaxanthin, and astaxanthin with DP = 20 were selected as representative additive samples. Castor oil (CastO) was selected as the lubricant base, because it is one of the most representative vegetable oils for the manufacturing of bio-lubricant products.^31^ Each polymer (0.5% weight) was diluted in castor oil at 70 °C. This mixture was stirred during 5 minutes until completely homogeneous lubricating solutions were obtained. The physical properties of bio-lubricant mixtures such as density, viscosity, and viscosity index were determined by the ASTM D891, ASTM D445, and ASTM D341 standards methods, respectively. Likewise, their friction and wear performance were evaluated by pin-on-disk tests based on the ASTM G 99 procedure. The performance of the polyester derived from 1,8-octanediol and CL (1,8-octanediol-PCL) with DP = 20 was also studied for comparison purposes. During experiments, friction coefficient was recorded by the equipment, while wear was evaluated at the end of the tests by calculating the wear rate.^32^

## Results and discussion

**Scheme 2:**
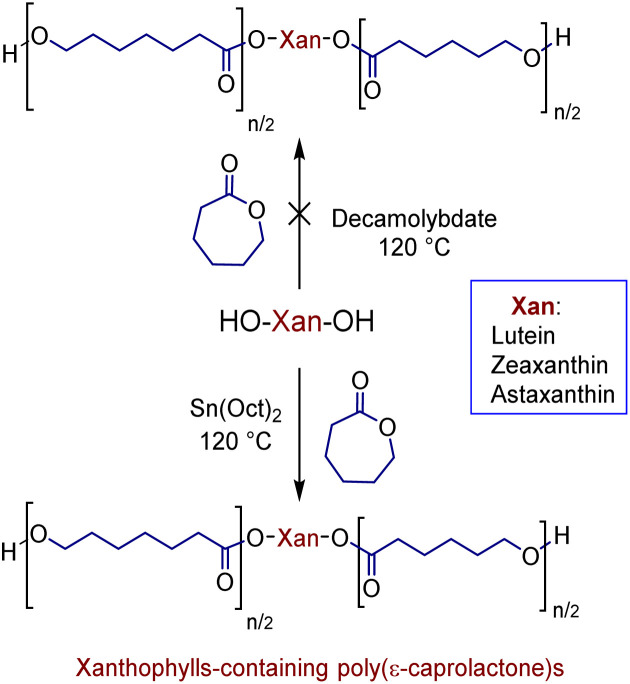
Synthesis of xanthophylls-containing poly(ε-caprolactone)s.

In previous studies, we have shown that the ammonium decamolybdate, (NH_4_)_8_[Mo_10_O_34_], is an excellent catalyst for ring-opening polymerization (ROP) of ε-caprolactone (CL).^33–37^ However, this catalyst is not efficient for the ROP when the xanthophylls are used as initiators, since the reaction did not occur (Scheme 2). This inefficiency is probably caused by the formation of other products. To confirm this assumption, the reaction between lutein and the ammonium decamolybdate was carried out in DCE as solvent at 80 °C for 24 hours (ES1). The reaction was monitored through reverse phase HPLC and the results indicated the presence of less polar products [probably dehydration products (anhydroluteins)] in the chromatogram (Fig. S2†). For this reason, we used the industrially relevant and accepted by the U.S. FDA^38,39^ catalyst, Sn(Oct)_2_, to obtain all xanthophylls-containing poly(ε-caprolactone)s (Scheme 2).

With the use of this catalyst, a solvent-free polymerization and at 120 °C, we synthesized three different macrodiols derived from each xanthophyll (1, 2, and 3) and CL with degrees of polymerization (DP) of 10, 20, and 40. For comparison, three different macrodiols were synthesized using 1,8-octanediol (Oct) as initiator, CL, and (NH_4_)_8_[Mo_10_O_34_] as catalyst with the same DPs (10, 20, & 40) [1,8-octanediol-PCL].

**Fig. 2:**
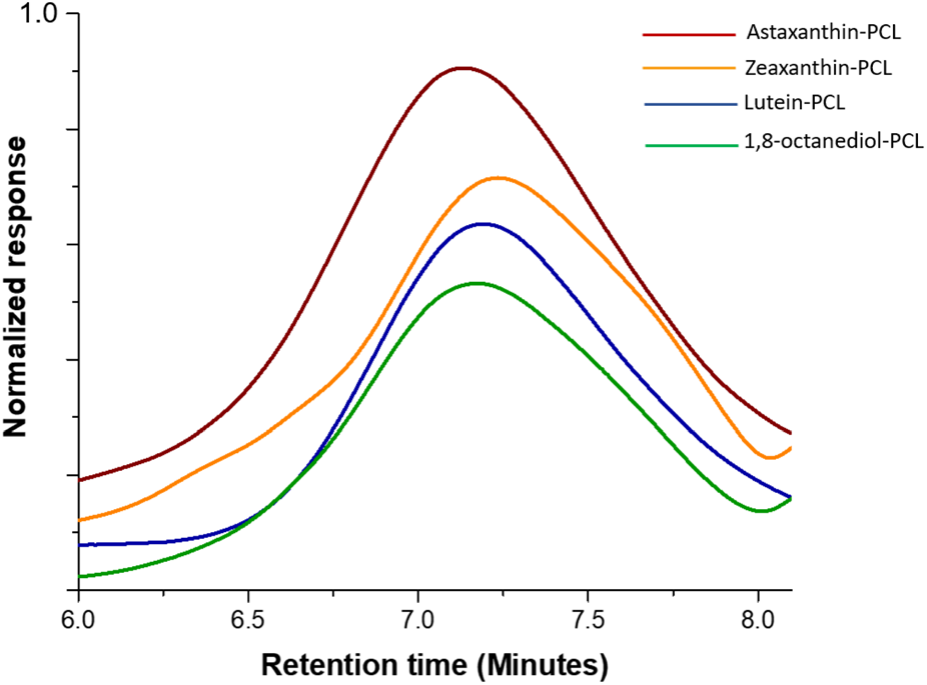
Refractive index-detected SEC chromatograms of the xanthophylls-PCL and 1,8-octanediol-PCL with DP = 20.

The polyesters were obtained with high conversions (from 94 to 99%). The time of reactions for these xanthophyll-PCLs varied from 20 to 48 hours, except those macrodiols synthesized using (NH_4_)_8_[Mo_10_O_34_] as catalyst (Table 1). The molecular weights for these macrodiols were determined by size-exclusion chromatography (SEC) analysis (Fig. 2) to be between 2500 and 10 500 Da (Table 1), where an unimodal distribution was observed.

**Table 1:** Results obtained in the polyesters synthesis from CL and xanthophylls

[M]/[I]^a^	[I]^b^	DP^c^	*M*_n_ d	*Đ*_M_ d ^,^ e	Time^f^ (hours)	Conversion^c^ (%)
10	1	11.0	3803	1.85	44.00	96
20		19.6	5361	1.67	48.00	94
40		40.0	10 365	1.58	48.05	94
10	2	10.0	4716	1.28	24.10	97
20		19.8	5000	1.24	24.00	97
40		40.0	8023	1.48	24.00	99
10	3	10.0	4927	1.38	20.00	99
20		20.0	5427	1.39	20.50	99
40		41.2	8466	1.46	20.05	99
10^g^	Oct	9.9	2767	1.65	2.08	99
20^g^		19.4	4978	1.41	2.14	97
40^g^		40.0	9134	1.40	2.05	98

a [Monomer] : [initiator] ratio.

b [I]: initiator.

c Determined by ^1^H NMR.

d Determined by SEC analysis.

e *Đ*_M_: dispersity.

f Time of reaction.

g Synthesized using ammonium decamolybdate as catalyst.

The xanthophyll-polyesters showed major dispersity (*Đ*_M_) between 1.24 & 1.85, compared to polymers derived from 1,8-octanediol, which are on average of *Đ*_M_ = 1.48 (Table 1). Therefore, the effect of the primary *versus* secondary alcohols (1,8-octanediol and xanthophylls, respectively) on the *Đ*_M_ was negligible. On the other hand, with regard to the reactivity, we confirmed that, among the three xanthophylls, astaxanthin is the most reactive for ROP, followed by zeaxanthin and finally lutein, based on their percentages of conversion obtained by ^1^H NMR and the time of reaction. This behavior in the reactivity of compound 1 is probably due to the less reactive allylic alcohol in the 3′ position, compared with the secondary alcohols in the zeaxanthin and astaxanthin.

**Fig. 3:**
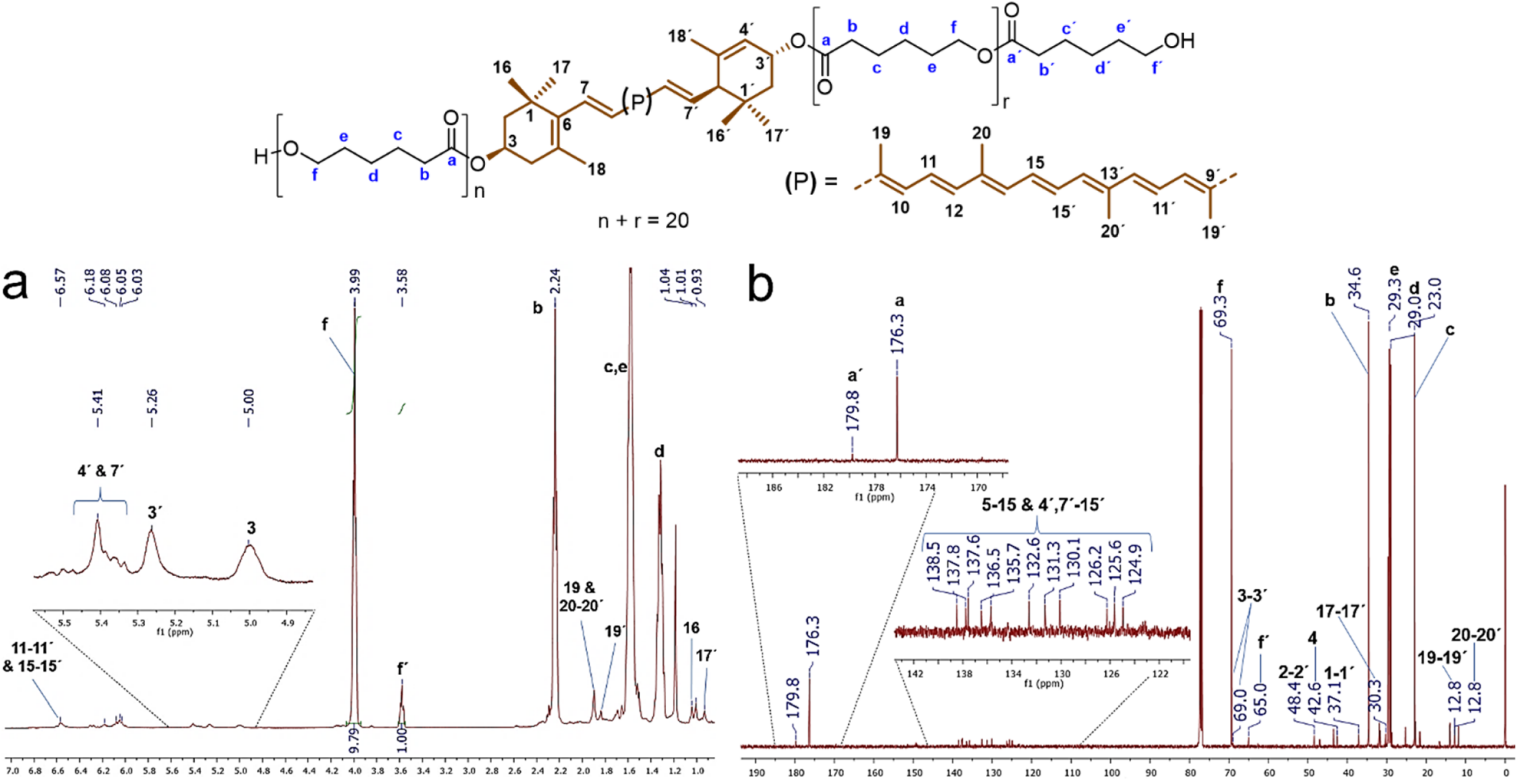
(a) ^1^H NMR (CDCl_3_, 500 MHz) and (b) ^13^C NMR (CDCl_3_, 125 MHz) spectra of polyester (DP = 19.6) derived from lutein (1).

Analysis by ^1^H and ^13^C NMR confirmed the presence of the xanthophylls in the structure of the polyesters. Fig. 3a shows the ^1^H NMR spectrum of the polyester derived from compound 1, lutein-poly(ε-caprolactone) diol (lutein-PCL), which indicates the characteristic signals for the oxygenated methylene (–CH̲_2_–O–) in the repetitive unit (f, *δ* 3.99) and the terminal hydroxymethylene (f', *δ* 3.58, –CH̲_2_–OH) of the PCL. The ratio of peak *f* area to that of peak *f*' is 9.8 : 1, which confirms that the main reaction product is HOPCLOH. This ratio multiplied by two indicates the degree of polymerization (DP), in this case, 19.6. The most intense signals were assigned to PCL: 2.24 ppm (b, –CH̲_2_–CO–O–), 1.58 ppm (c & e, –CH_2_–), and 1.30 ppm (d, –CH_2_–). This spectrum also revealed a shift of resonances corresponding to the lutein protons upon poly(ε-caprolactone) chain growth, most notably in the chemical shift attributed to the protons attached to the 3 & 3′ carbons from 4.01 and 4.25 (Fig. S3†) to 5.00 and 5.26 ppm upon polymerization, respectively. The same effect was observed in the macrodiols derived from compounds 2 and 3 (Table S2†). The protons 3 & 3′ in the ^1^H spectrum of zeaxanthin appears from 4.01 ppm (Fig. S4†) to 5.00 ppm (Fig. S6†) upon polymerization. While in the ^1^H spectrum of astaxanthin-poly(ε-caprolactone) homopolymer (astaxanthin-PCL) was observed from 4.33 ppm (free astaxanthin, Fig. S5†) to 5.34 ppm (Fig. S7†). Finally, in the ^1^H NMR spectrum of lutein-PCL, some signals, such as vinylic protons between 6.57 and 6.03 ppm and those singlets of the methyl groups at 1.97 ppm (19, 19′, 20, & 20′) and 1.07 ppm (16, 16′, 17, & 17′) corresponding to the xanthophyll moiety were assigned.

In the same way, the ^13^C spectrum also shows evidence for the presence of xanthophyll in the structure of the polymer. Fig. 3b shows the ^13^C spectrum of the macrodiol derived from compound 1 with DP = 20. The signals at 176.3 and 179.8 ppm correspond to the ester carbonyl group of the main chain (a) and the ester carbonyl of the end group (a'), respectively. Signals at 138.5 to 124.9 ppm correspond to vinylic carbons of the xanthophyll moiety (5–15 & 4′, 7′–15′) of the PCL. Meanwhile the signal at *δ* 69.3 (f, –C̲H̲_2_–O–CO–) was assigned to the methylene attached to the sp^3^ oxygen of the ester group and the signal at *δ* 34.6 (b, –C̲H̲_2_–CO–O–) is ascribed to the methylene bound to the ester group. The other most intense peaks at 29.3 ppm (e, –CH_2_–), 29.0 ppm (d, –CH_2_–), and 23.0 ppm (c, –CH_2_–) observed in the spectrum were assigned to the other methylenes of PCL. In addition, the signals corresponding to the lutein moiety, such as those of the methylenes at 48.4 and 42.6 ppm corresponding to carbons 2, 2′ and 4, respectively, as well as methyl groups at *δ* 30.3 (17,17′) and 12.8 (19,19′ & 20,20′), and that methines 3 and 3′ at *δ* 69.0 and 69.3 were identified, respectively.

**Fig. 4:**
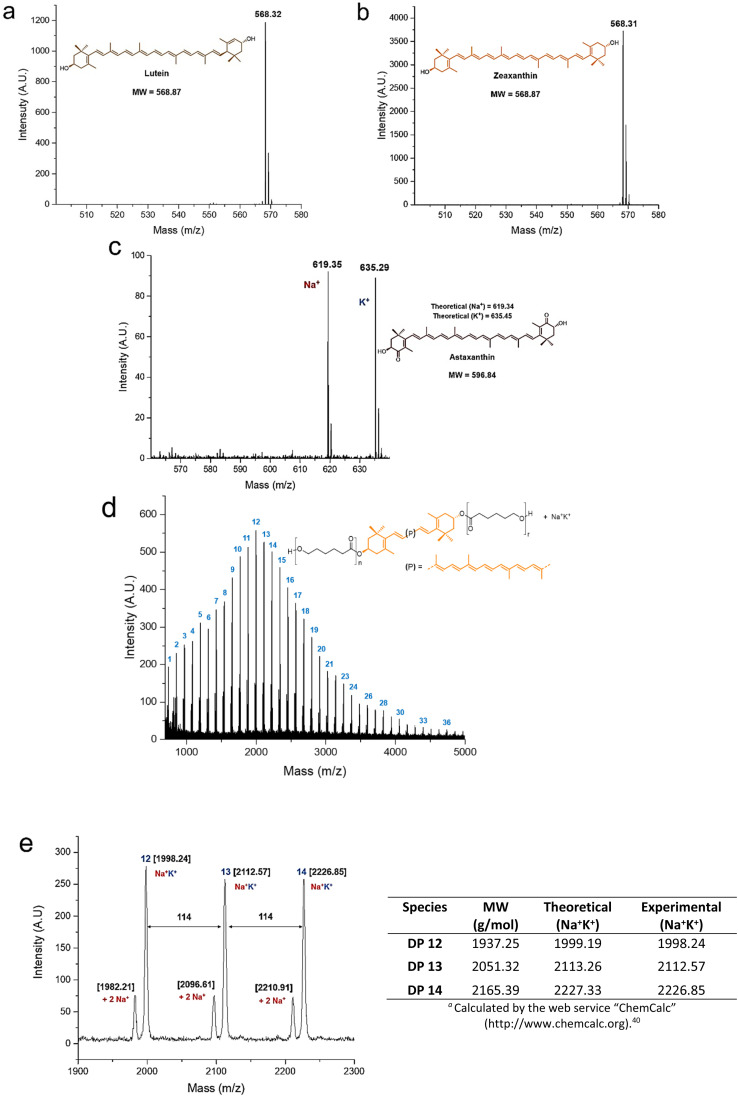
MALDI-TOF mass spectra (linear mode) of the three initiators: (a) lutein, (b) zeaxanthin, and (c) astaxanthin (doped with Na^+^ and K^+^ ions); (d) mass spectrum of polyester derived from zeaxanthin, the number indicates the degree of polymerization (DP) of each peak, and (e) expanded view for the 1900–2300 fragments (12–14 CL repeating units) doped with two Na^+^ and Na^+^–K^+^ ions; 114 is the molecular weight of the CL monomer.

The MALDI-TOF analysis provided useful information for the characterization of the polyesters and their initiators (Fig. 4). The xanthophylls 1 and 2 were shown as molecular ion species (Fig. 4a and b), while compound 3 is observed doped with sodium and potassium ions (Fig. 4c). In the MALDI-TOF mass spectrum for zeaxanthin-poly(ε-caprolactone) diol with degree of polymerization equal to 10 (Fig. 4d) it was clearly observed the characteristic pattern of the curve profile with a unimodal molecular weight distribution of a PCL oligoester with a specific DP. Notably, the major repeat unit is 114 Da corresponding to CL unit. In an expanded view of the MALDI-TOF mass spectrum of zeaxanthin-PCL with DP = 10 (Fig. 4e), fragments between 1900 and 2300 Da are observed; in this zoom, the oligomers with 12–14 CL repeat units are visualized. The most intense peaks are doped with sodium and potassium ions (Na^+^K^+^), while low-intensity peaks are doped with two sodium ions (2Na^+^).

This pattern also was observed in other samples (Fig. S11†).^40^ On the other hand, a previous report about “retinol initiated poly(lactide)s” has described the presence of epoxide groups as a consequence of oxidation in the structure of retinoid.^41^ This might occur in the xanthophylls-PCL. However, the MALDI–TOF analysis of our samples did not reveal the presence of a mixture of natural and epoxidized xanthophylls (Fig. S14†), as it was described in the poly(lactide)s derived from retinol. Finally, it is worthy to mention that the presence of two sodiums or sodium-potassium adducts was not observed in the polyesters derived from 1,8-octanediol (Fig. S13†). Additionally, mass signals for macrocyclic species are not visualized, which indicates that intramolecular transesterification reactions neither occur. Take into consideration that xanthophylls are susceptible to epoxidations, this particularity can be used to increase the functionality of these polymers, which eventually we will describe in a future contribution (Scheme S1†).

**Fig. 5:**
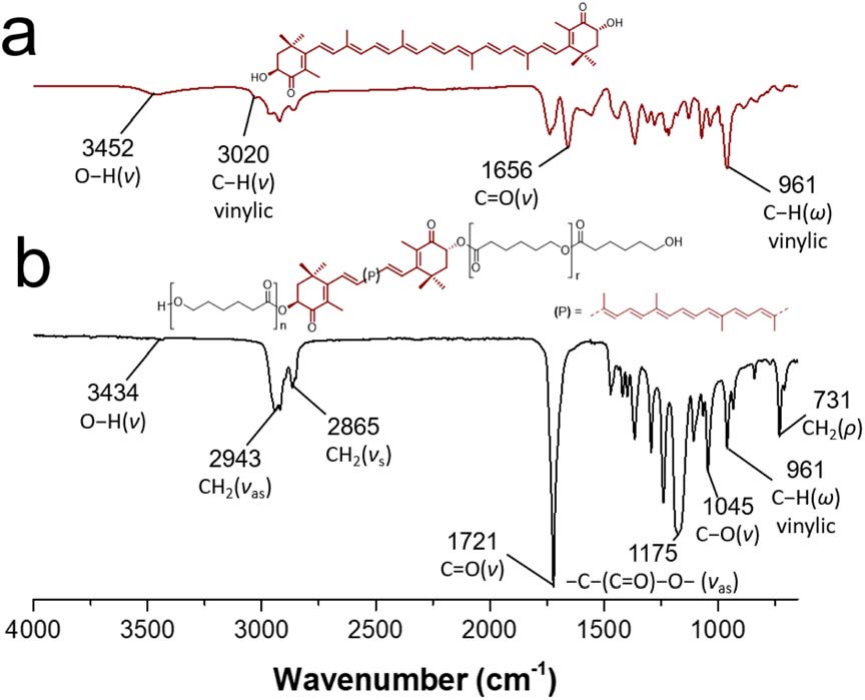
FT-IR spectrum of (a) free astaxanthin and (b) astaxanthin-poly(ε-caprolactone) homopolymer (DP = 20).

In the same way, FTIR-ATR spectroscopy was useful for the characterization of the macrodiols. Fig. 5b shows the FT-IR spectrum of astaxanthin-PCL, where it can be observed the out-of-plane C–H bending vibration band at 961 cm^−1^ [(C=C–H)] (*ω*), characteristic of *trans*-disubstituted alkenes, which corroborated the xanthophyll insertion. Likewise, the typical stretching vibrations of –CH_2_– (*ν*_as_ and *ν*_s_) were shown at 2943 and 2865 cm^−1^, respectively. Other signals at 1175 and 731 cm^−1^ were assigned to stretching vibration of [–C–(C=O)–O–] and bending vibration of (–CH_2_–) (rocking, *ρ*). With regard to the xanthophyll 3 used as initiator (Fig. 5a), a broad band at 3452 cm^−1^ was attributed to the hydroxyl groups and another thin band at 3020 cm^−1^ to the C–H stretching of the vinylic group. Additionally, the ketone carbonyl band at 1656 cm^−1^ and the stretching vibrations of methylene groups (–CH_2_–) were observed at 2859 (*ν*_s_) and 2921 (*ν*_as_) cm^−1^. These data match those published by others.^42,43^

Moreover, the thermal properties, such as melting temperature (*T*_m_) and crystallinity (*χ*_i_), of all polyesters were examined by differential scanning calorimetry (DSC). Table 2 summarizes the melting temperatures of all polymers which were observed between 36.1 and 53.5 °C. There is a clear correlation in the increase of the *T*_m_ as DP increases. The degree of crystallinity for all polymers was calculated from the endothermic peak area Δ*H*_i_ by the equation *χ*_i_ = Δ*H*_i_/Δ*H*^°^_i_, where Δ*H*^°^_i_ is the heat of fusion (135.3 J g^−1^, perfect PCL crystal).^44^

**Table 2:** Thermal properties of different xanthophylls-PCL

Initiator	DP^a^	*T*_m_ b (°C)	Δ*H*_m_^b^ (J g^−1^)	*χ*_i_ c (%)
Lutein	11.0	40.0, 44.7	56.77	42
	19.6	47.1, 49.7	65.57	48
	40.0	51.3, 53.0	74.77	55
Zeaxanthin	10.0	43.5, 47.9	70.23	52
	19.8	48.0, 50.2	74.82	55
	40.0	51.7, 53.5	77.97	58
Astaxanthin	10.0	—	—	—
	20.0	46.77	56.39	42
	41.2	50.01	66.94	49
1,8-Octanediol	9.9	36.1, 41.0	84.62	62
	19.4	44.3, 46.8	75.87	56
	40.0	50.1, 52.3	77.73	57

a Determined by ^1^H NMR.

b Obtained by DSC, second heating cycle.

c Obtained by the equation *χ*_i_ = (Δ*H*_i_/Δ*H*^°^_i_) (100).

**Fig. 6:**
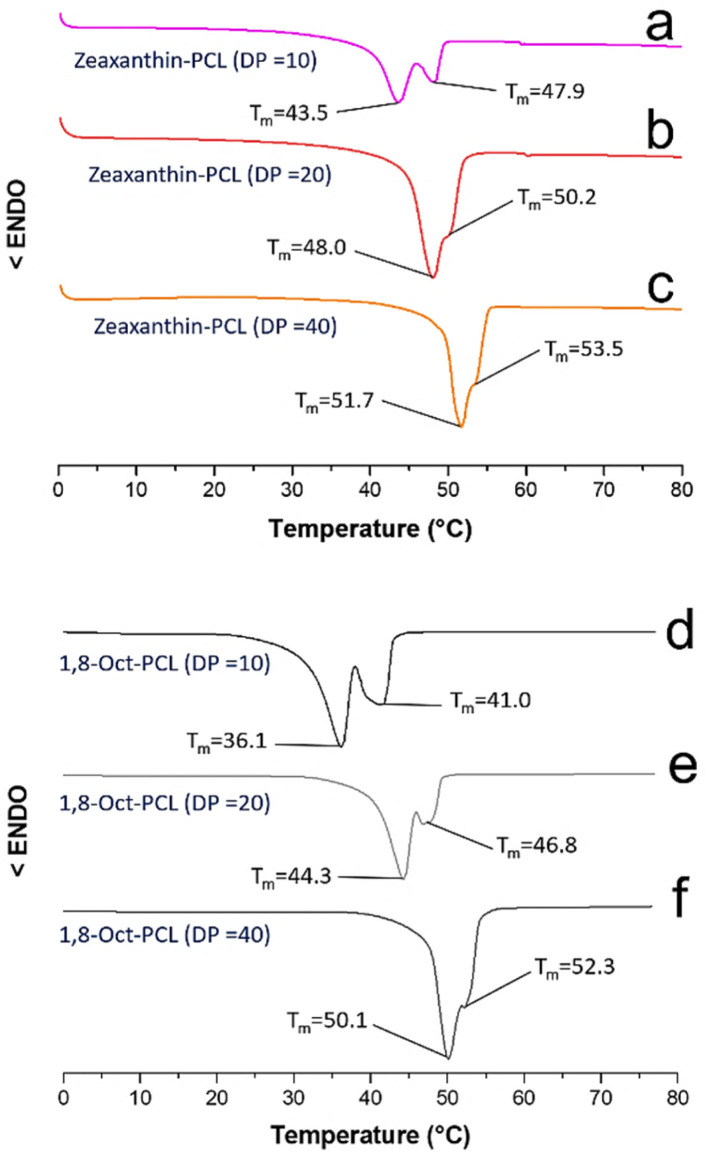
DSC thermograms of the three different macrodiols derived from zeaxanthin (a–c) and the macrodiols derived from 1,8-octanediol (d–f). Conditions: 10 °C min^−1^ (endo down); second heating cycle.

Fig. 6 shows the DSC thermograms of the macrodiols derived from the zeaxanthin and those derived from 1,8-octanediol with DP = 10, 20, and 40.

The melting temperatures of the polyesters derived from compound 2 were detected between 43.5 and 53.5 °C (Fig. 6a–c). In the thermogram of zeaxanthin-PCL with DP = 10 (Fig. 6a) two endothermic peaks (*T*_m_ = 43.5 and 47.9 °C) were observed. The two signals may be explained in terms of two different sizes of crystallites that arise from two different environments, for example, crystallites immersed in an amorphous phase (43.5 °C) and in zones that are less amorphous in the PCL (47.9 °C). Similar behaviour in the 1,8-Oct-PCL with DP = 10 (Fig. 6d) was observed.

While the *T*_m_ of the 1,8-octanediol-PCL was observed between 36.1 and 52.3 °C (Fig. 6d–f). Generally, the polymers derived from 1,8-octanediol have a slightly lower *T*_m_ than xanthophylls-PCL. These results may be attributed to the significant crystalline domains in the xanthophylls, acting as nucleation agents (Fig S15†). The *T*_m_ of xanthophylls-PCL are near 45 °C, which is consistent with PCL oligoesteres previously reported.^45^ A single endothermic peak (*T*_m_) was observed for astaxanthin-PCL samples (Fig. S16†). All the polyesters show a tendency towards increased crystallinity with increasing DP.

Table 3 summarizes the physical properties, as well as the friction and wear performance of bio-lubricants. The density of the bio-lubricants was not significantly affected with the addition of the polyesters. However, the viscosity and viscosity index were influenced by the type of initiator. In comparison with 1,8-octanediol-PCL, lutein-PCL allowed to increase the viscosity and the viscosity index of bio-lubricants up to 7% and 15%, respectively. Zeaxanthin-PCL decreased the viscosity by 12%, but increased the viscosity index by 26%. However, astaxanthin-PCL decreased both the viscosity and viscosity index to 5% and 26%, correspondingly. Regarding the effect these additives produced on the friction and wear performance, it can be observed that the lowest friction coefficient value was achieved with the additive containing lutein-PCL. With this component, the friction coefficient was 50% lower than that obtained with 1,8-octanediol-PCL. Nevertheless, an increase in friction of 28% was observed with astaxanthin-PCL. On the other hand, the wear rate was notably improved by all the xanthophylls-PCL. The higher contribution was observed with zeaxanthin-PCL, whose wear rate value was up to 39% lower than that acquired with 1,8-octanediol-PCL.

**Table 3:** Physical properties and tribological performance of bio-lubricants

Bio-lubricant	Density^a^ (Kg m^−3^)	Kinematic viscosity^a^ (mm^2^ s^−1^)	Viscosity index	FC^b^	Wear rate^b^ (mm^3^ Nm^−1^)
CastO – 0.5% wt lut-PCL	943	286	153	0.07	1.45 × 10^−5^
CastO – 0.5% wt zea-PCL	944	236	167	0.12	1.24 × 10^−5^
CastO – 0.5% wt ast-PCL	951	254	98	0.18	1.71 × 10^−5^
CastO – 0.5% wt 1,8-PCL	942	267	133	0.14	2.02 × 10^−5^

a Properties at 40 °C.

b Friction and wear test employing tungsten carbide pins of 3 mm, steel disks of 25.4 mm, 60 mL of bio-lubricant at 100 °C, normal load of 1 N, sliding speed of 0.025 m s^−1^, and total sliding distance of 377 m.

**Fig. 7:**
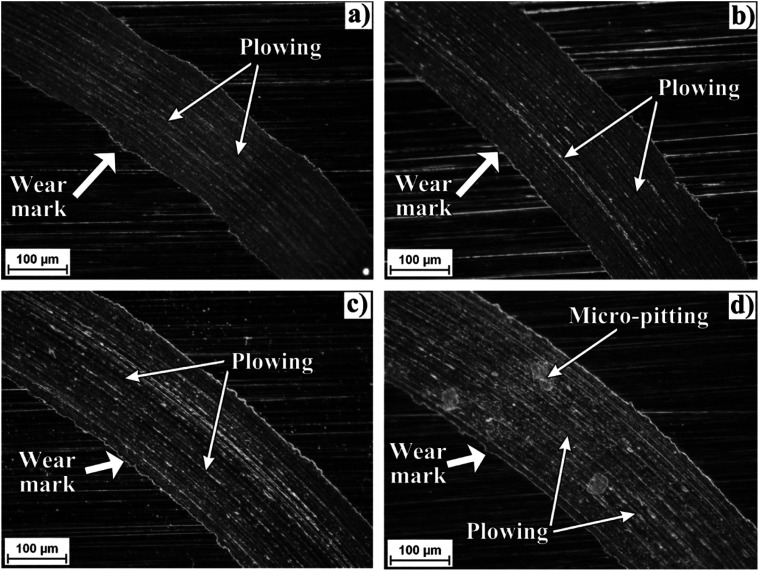
Optical micrograph at 200× of a region of the disk surface tested with castor oil–0.5% wt lutein-PCL (a), with castor oil–0.5% wt zeaxanthin-PCL (b), with castor oil–0.5% wt astaxanthin-PCL (c), and with castor oil–0.5% wt 1,8-octanediol-PCL (d).

Fig. 7 shows an optical micrograph of a region of the disk surfaces tested with the studied bio-lubricants. Within the wear track marks, it can be observed that wear damage was different depending on the bio-lubricant employed. Worn surfaces lubricated with castor oil and xanthophylls-PCL exhibited only plowing wear which is characterized by the formation of grooves and furrows. Meanwhile, the surface tested with the bio-lubricant with 1,8-octanediol-PCL, besides showing wider and deeper grooves, exhibited pitting marks which are a form of localized corrosion that leads to the creation of small holes in the metal. In this sense, the intrinsic antioxidant activity of carotenoids into the xanthophylls-PCL could be contributed to the diminishing corrosion process. In a previous contribution, we demonstrated that xanthophylls 1, 2, and 3 are excellent additives for totally biodegradable lubricants, because they improve the tribological and antioxidant properties.^32^ In this context, the xanthophylls-PCL could be also employed with this purpose. The best results obtained in this work are the samples that contain lutein and zeaxanthin homopolymers may be due to the improvement in viscosity and viscosity index, which is a scale used to measure the change of the resistance to flow of a lubricant with the increase of its temperature. Furthermore, the greater the viscosity, the greater the layer that separates the mechanical elements, thus reducing friction and wear between them.

Finally, different studies have reported the use of carotenoids as stabilizers for both polymers and biopolymers to improve the resistance to the factors that promote their deterioration such as UV light, oxygen, heat, among others.^46^ Eventually, our group will report in a future contribution the properties of these xanthophylls-PCL and the free carotenoids as additives (compatibilizers, plasticizers, and antioxidants) in polymeric blends for the development of food packaging with antioxidant properties.

## Conclusions

Xanthophylls-containing poly(ε-caprolactone)s were successfully synthesized by solvent-free ring-opening polymerization (ROP) of ε-caprolactone (CL), tin(ii) octoate Sn(Oct)_2_ as catalyst, and using three different xanthophylls, (3*R*,3′*R*,6′*R*)-lutein (1) extracted from a renewable source (marigold oleoresin, *Tagetes erecta* L.), (3*R*,3′*S*)-zeaxanthin (2) and (3*R*,3′*S*)-astaxanthin (3) obtained by semisynthesis from 1, as initiators. Xanthophylls incorporation into the structure of the polyesters were confirmed by ^1^H and ^13^C NMR, MALDI-TOF mass spectrometry, and FT-IR spectroscopy analysis. Thermal properties of xanthophyll-polyesters showed a crystalline domains and melting temperatures between 36.1 and 53.5 °C, characteristic of classical PCL homopolymer, detected by DSC.

All xanthophylls-PCL showed a better tribological behavior than current additives, which demonstrates their potential as future commercial materials with antioxidant and eco-friendly properties for diverse applications ranging from additives in green lubricants, antioxidant coatings in metallic surfaces for corrosion prevention, as additives for manufacturing of food packaging materials with antioxidant capacity, among others. Finally, this work represents the first report on the use of xanthophylls as initiators in the ring-opening polymerization of ε-caprolactone.

## Conflicts of interest

There are no conflicts to declare.

## Supplementary Material

RA-012-D2RA04502H-s001
