# Supplementary material for: Sustainable xanthophylls-containing poly(ε-caprolactone)s: synthesis, characterization, and use in green lubricants
Source: RSC Adv. 2022 Oct 27;12(48):30851–9. doi: 10.1039/d2ra04502h (PMC9609694; doi:10.1039/d2ra04502h)
Supplement: RA-012-D2RA04502H-s001 [file RA-012-D2RA04502H-s001.pdf]

Electronic Supplementary Material (ESI) for RSC Advances.
This journal is © The Royal Society of Chemistry 2022

Supplementary Information

Sustainable xanthophyll-containing poly(ε-caprolactone)s: synthesis, characterization, and its use in green lubricants

Eloy Rodríguez-deLeón,[a*] Moustapha Bah,[a] José E. Báez,[b*] María T. Hernández-Sierra,[c] Karla J. Moreno,[c] Alejandro Nuñez-Vilchis,[a] José Bonilla,[d] and Kenneth J. Shea[e]

[a] Posgrado en Ciencias Químico Biológicas, Faculty of Chemistry, Autonomous University of Queretaro, Cerro de las Campanas 76010, Queretaro, Mexico.

[b] Department of Chemistry, Division of Natural and Exact Sciences, University of Guanajuato, Noria Alta s/n 36050, Guanajuato, Mexico.

[c] Department of Mechanical Engineering, National Technology of Mexico at Celaya, Celaya 38010, Guanajuato, Mexico.

[d] Centro de Investigación en Materiales Avanzados S.C. (CIMAV-Unidad Monterrey), Av. Alianza del Norte 202, PIIT, Autopista Monterrey-Aeropuerto Km 10 66628, Apodaca, N.L., Mexico.

[e] Department of Chemistry, University of California, Irvine, (UCI), Irvine, California 92697-2025, USA.

To whom correspondence should be addressed: Eloy Rodríguez-deLeón, e-mail: eloy.rodriguez@uaq.mx and José E. Báez, e-mail: jbaez@ugto.mx

Table of Contents

		Page
**Experimental**		**4**
	**ES1.** Reaction between lutein and ammonium decamolybdate.	4
	**ES2.** HPLC analysis.	4
	**Table S1.** Gradient used for the analysis of carotenoid conversions.	4
**HPLC chromatograms**		**5**
	**Fig S1.** HPLC chromatogram of free lutein.	5
	**Fig S2.** HPLC chromatogram of the crude reaction product between lutein and ammonium decamolybdate.	6
**^{1}H NMR spectra of Xanthophylls**		**7**
	**Fig. S3.** ^{1}H NMR spectrum of (3*R*,3´*R*,6´*R*)-Lutein (500 MHz, $CDCl_3$).	7
	**Fig. S4.** ^{1}H NMR spectrum of (3*R*,3´*S*)-Zeaxanthin (500 MHz, $CDCl_3$).	8
	**Fig. S5.** ^{1}H NMR spectrum of (3*R*,3´*S*)-Astaxanthin (500 MHz, $CDCl_3$).	9
**^{1}H NMR spectra of Polyesters**		**10**
	**Fig. S6.** ^{1}H NMR spectrum of macrodiol (DP = 40) derived from zeaxanthin (500 MHz, $CDCl_3$).	10
	**Fig. S7.** ^{1}H NMR spectrum of (3*R*,3´*S*)-Astaxanthin (500 MHz, $CDCl_3$).	11
	**Table S2.** Chemical shift in ^{1}H NMR spectrum of the 3 & 3´protons of the xanthophylls upon polymerization.	11
	**Fig. S8.** ^{1}H NMR spectrum of macrodiol (DP = 9.9) derived from 1,8-octanediol (500 MHz, $CDCl_3$).	12
**MALDI-TOF spectra**		**13**
	**Fig. S9.** Isotopic distribution calculated for **a** ($C_{40}H_{56}O_2$, lutein or zeaxanthin) and **b** ($C_{40}H_{52}O_4K^+$, astaxanthin doped with K^+) in “ChemCalc” (http://www.chemcalc.org).	13

	**Fig. S10.** MALDI-TOF spectrum of lutein-PCL.	14
	**Fig. S11.** Expanded view of the MALDI-TOF spectrum of lutein-PCL.	15
	**Table S3**. MW of the expanded view of MALDI-TOF spectrum of lutein–PCL with DP = 20.	15
	**Fig. S12.** MALDI-TOF spectrum of 1,8-octanediol-PCL.	16
	**Fig. S13.** Expanded view of the 950-1350 m/z fragments of Figure S12 MALDI-TOF spectrum for the 1,8-octanediol-PCL.	17
	**Table S4.** Expanded view of the MALDI-TOF mass spectrum of the 1,8-octanediol-PCL with DP = 10.	17
	**Fig. S14.** Tentative structures of “epoxy”-xanthophylls-PCL.	18
	**Scheme S1.** Functionalization of “epoxy”-xanthophylls-PCL.	18
**Thermal properties of PCL Macrodiols (HOPCLOH) obtained by DSC**		**19**
	**Fig. S15.** DSC thermograms (cooling, T_c) of **a**) 1,8-octanediol-PCL (DP = 40), and **b**) zeaxanthin-PCL (DP = 40).	19
	**Fig. S16.** DSC thermograms of astaxanthin-PCL.	19

Experimental

ES1. *Reaction between lutein and ammonium decamolybdate*. - Into a flask of 25 mL 288.5 mg of lutein (0.505 mmol), 3 mg of ammonium decamolybdate $(NH_4)_8[Mo_{10}O_{34}]$, and 12 mL of 1,2-dichloroethane (DCE) were placed. The mixture was heated to 80 °C for 24 hours and analyzed by reverse-phase HPLC every 30 minutes. 0.5 mL of a sample of the crude reaction product was collected and diluted in 10 mL of acetone. This solution was newly diluted in 10-fold acetone and filtered on acrodisc of nylon with 0.22 µm pore size.

ES2. *HPLC Analysis*. - HPLC analysis were carried out on a Waters equipment (Milford, MA, USA), composed of a 2695e quaternary pump and a 2998 diode array detector (DAD). The data acquisition was performed by the Empower 3 software (Waters®). Chromatographic conditions: *Column*: Develosil RP-aqueous C_{30}, 5µm, 140Å, 250 x 4.6 mm i.d.; *flow rate*: 0.7 mL/min; *detection at* λ = 450 nm; *injection volume*: 10 µL; *run time*: 35 minutes; *mobile phase*: gradient of methanol (MeOH)–methyl tert-butyl ether (MTBE)–H_2O (Table S1.).

Table S1. Gradient used during the analysis of carotenoid conversions.

Time (minutes)	MeOH (%)	MTBE (%)	H_2O (%)
**0**	81	15	4
**15**	66	30	4
**23**	16	80	4
**27**	16	80	4
**27.1**	81	15	4
**35**	81	15	4

HPLC chromatograms:

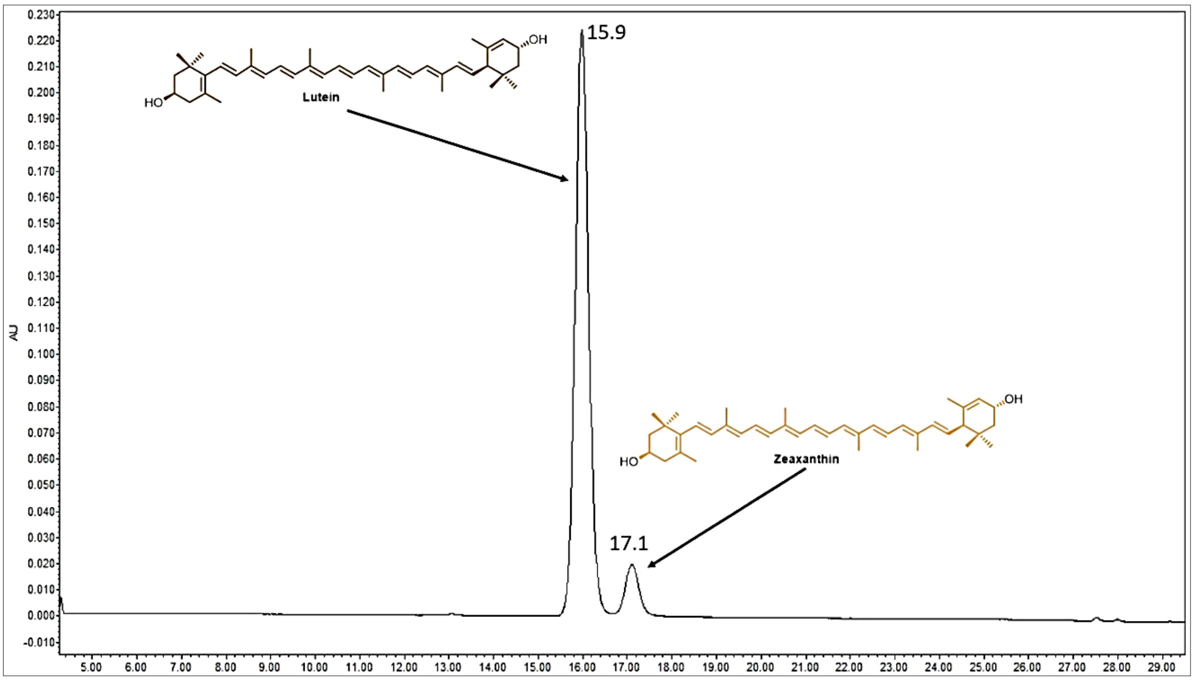

Fig S1. HPLC chromatogram of free lutein with retention time (TR) of 15.9 minutes. The peak at TR equal to 17.1 minutes corresponds to natural zeaxanthin present in the marigold oleoresin.

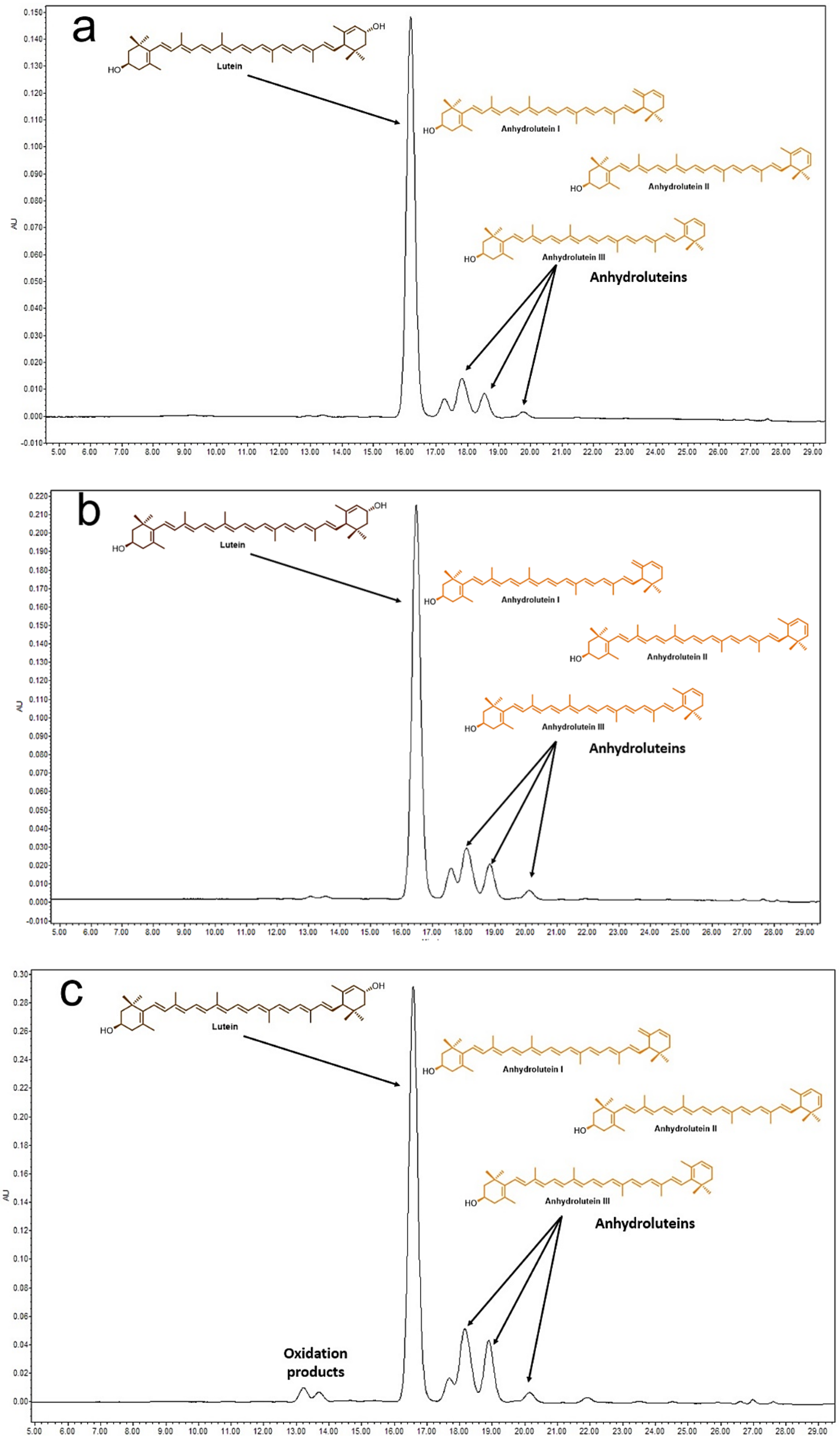

Fig S2. HPLC chromatogram of the crude reaction product between lutein and ammonium decamolybdate at 2 (a), 4 (b), and 24 hours (c). The peaks with the longer retention times (compounds less polar than lutein) indicate the presence of dehydration products.

^{1}H NMR spectra of Xanthophylls:

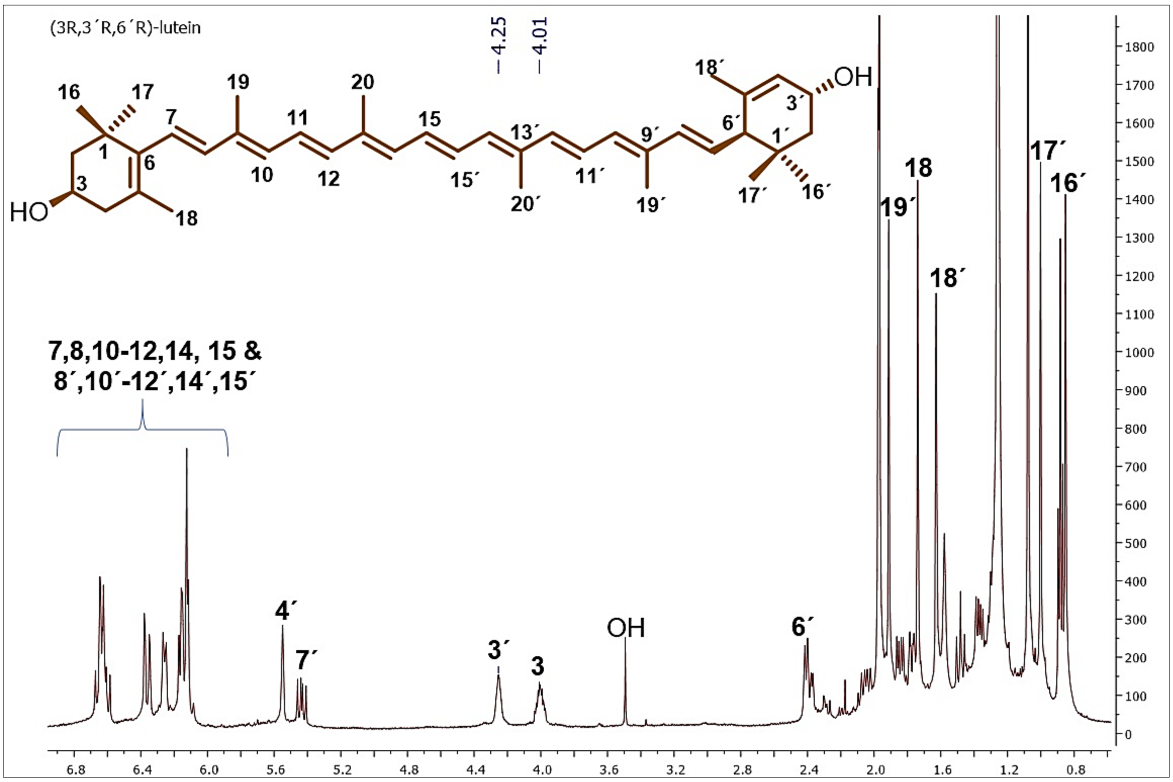

Fig. S3. ^{1}H NMR spectrum of (3*R*,3´*R*,6´*R*)-Lutein (500 MHz, $CDCl_3$).

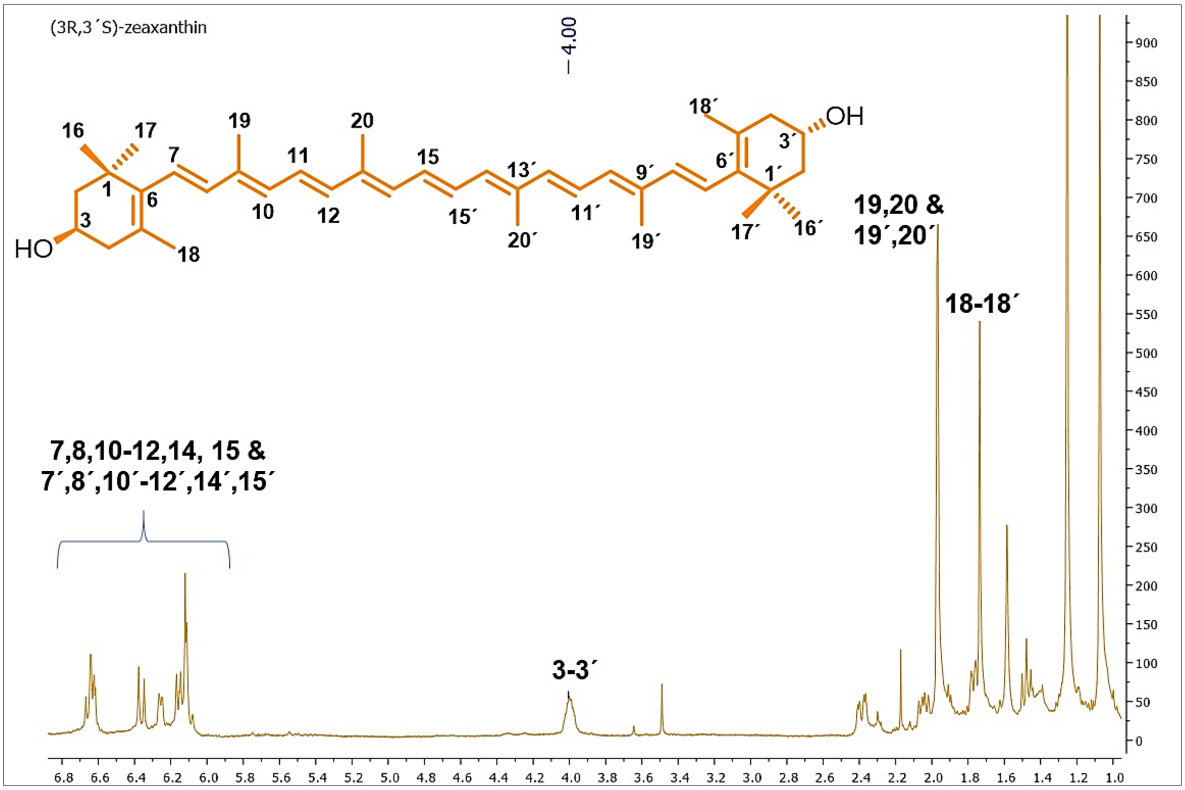

Fig. S4. ^{1}H NMR spectrum of (3*R*,3´*S*)-Zeaxanthin (500 MHz, $CDCl_3$).

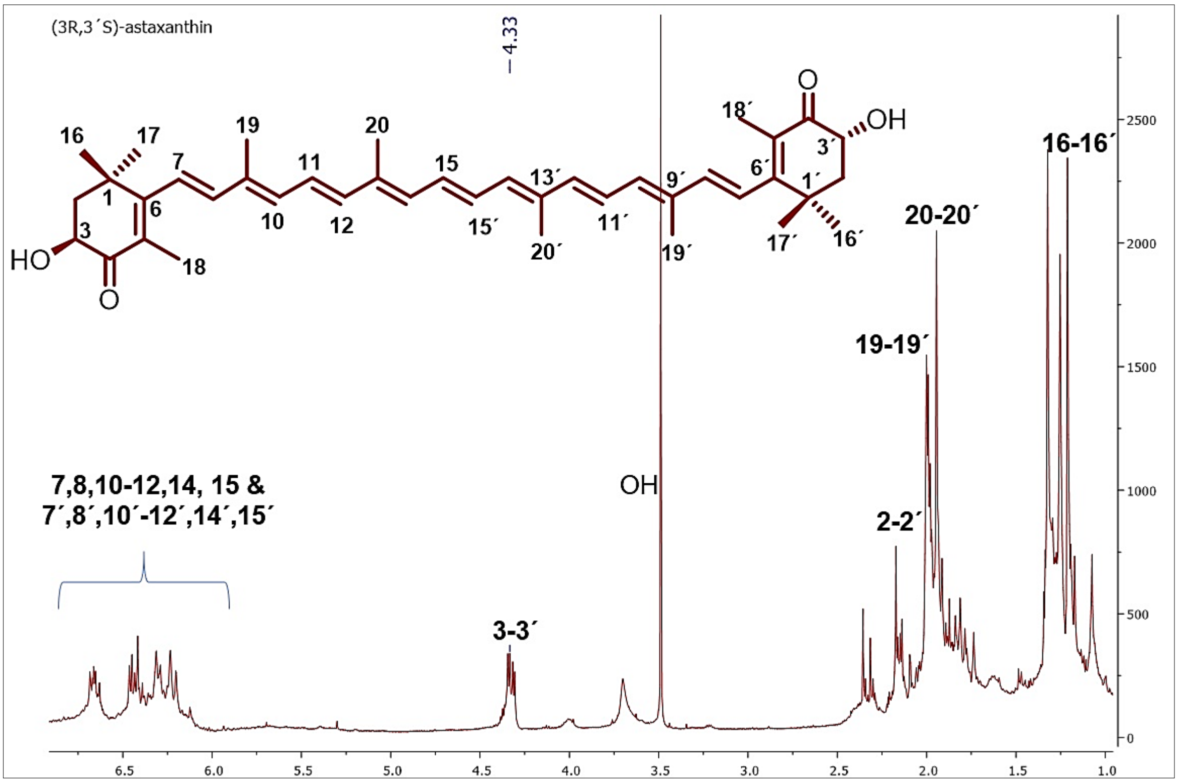

Fig. S5. ^{1}H NMR spectrum of (3*R*,3´*S*)-Astaxanthin (500 MHz, $CDCl_3$).

^{1}H NMR spectra of Polyesters:

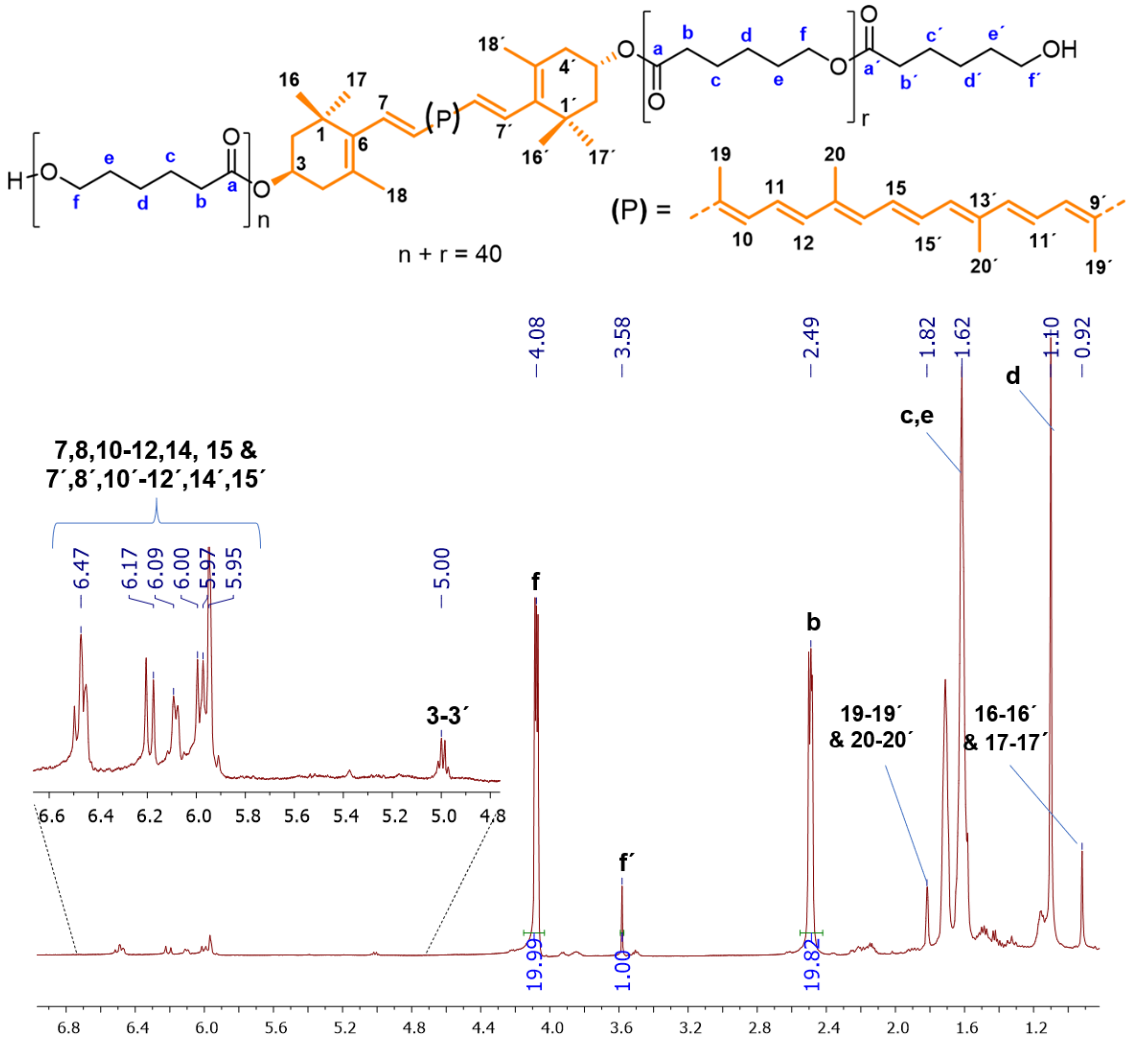

Fig. S6. ^{1}H NMR spectrum of macrodiol (DP = 40) derived from zeaxanthin (500 MHz, $CDCl_3$).

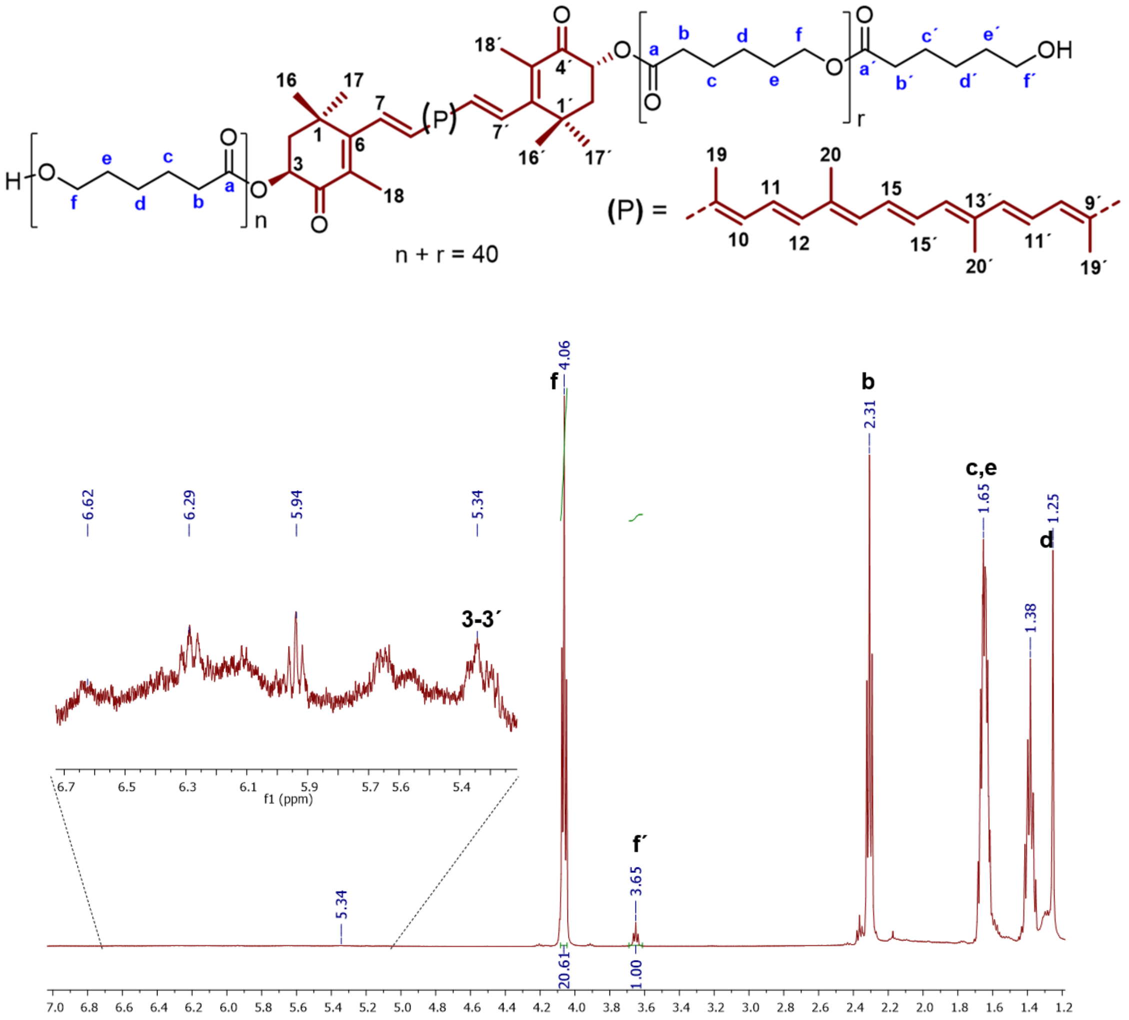

Fig. S7. ^{1}H NMR spectrum of macrodiol (DP = 41.2) derived from astaxanthin (500 MHz, $CDCl_3$).

Table S2. Chemical shift in ^{1}H NMR spectrum of the 3 & 3´protons of the xanthophylls upon polymerization.

Xanthophyll	Free Xanthophyll chemical shifts (ppm)		Xanthophyll-PCL chemical shifts (ppm)	
	H-3	H-3´	H-3	H-3´
(3*R*,3´*R*,6´*R*)-Lutein	4.01	4.25	5.00	5.26
(3*R*,3´*S*)-Zeaxanthin	4.00		5.00	
(3*R*,3´*S*)-Astaxanthin	4.33		5.34	

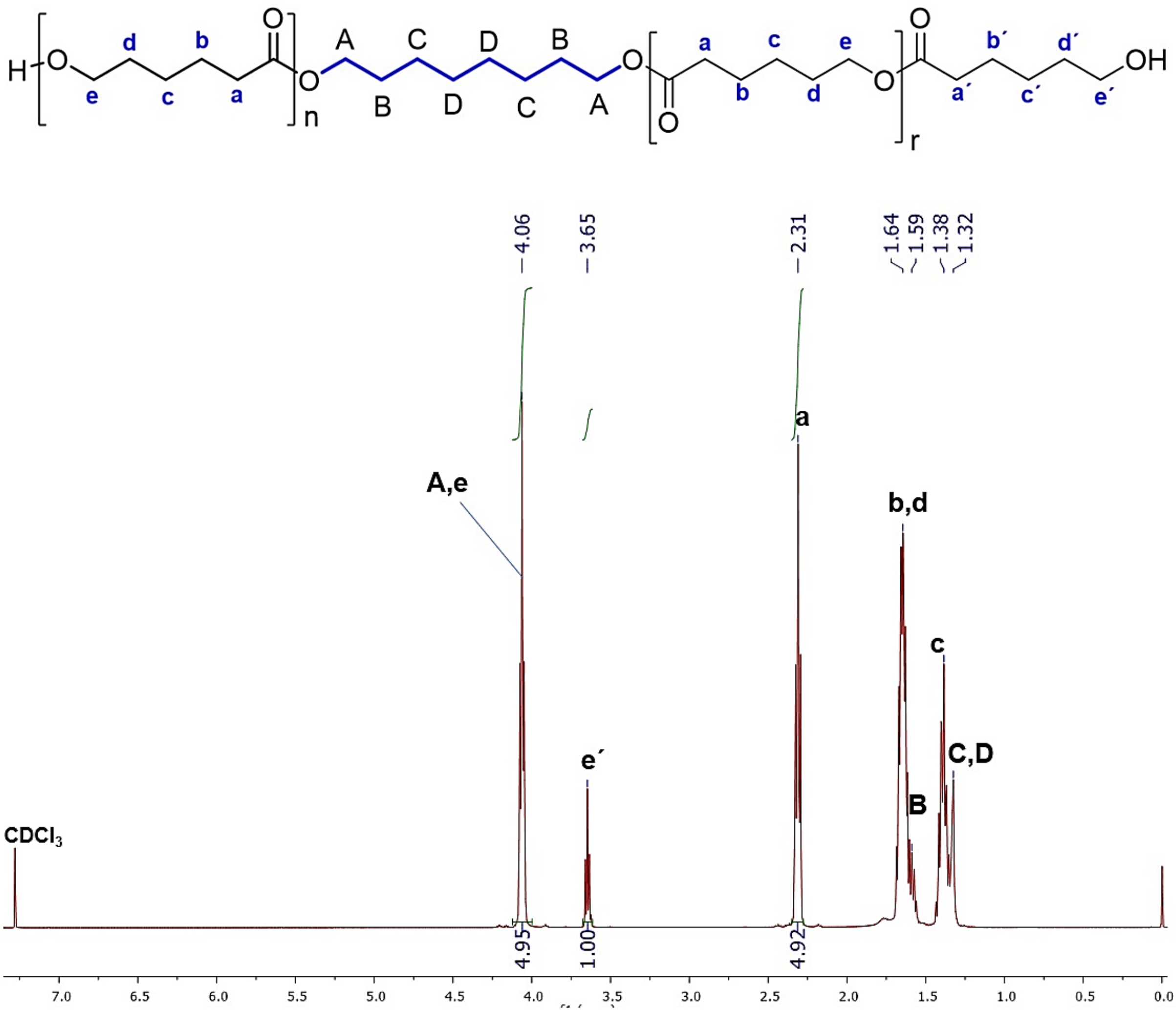

Fig. S8. ^{1}H NMR spectrum of HOPCLOH with DP = 9.9 (500 MHz, $CDCl_3$).

MALDI-TOF spectra of polyesters:

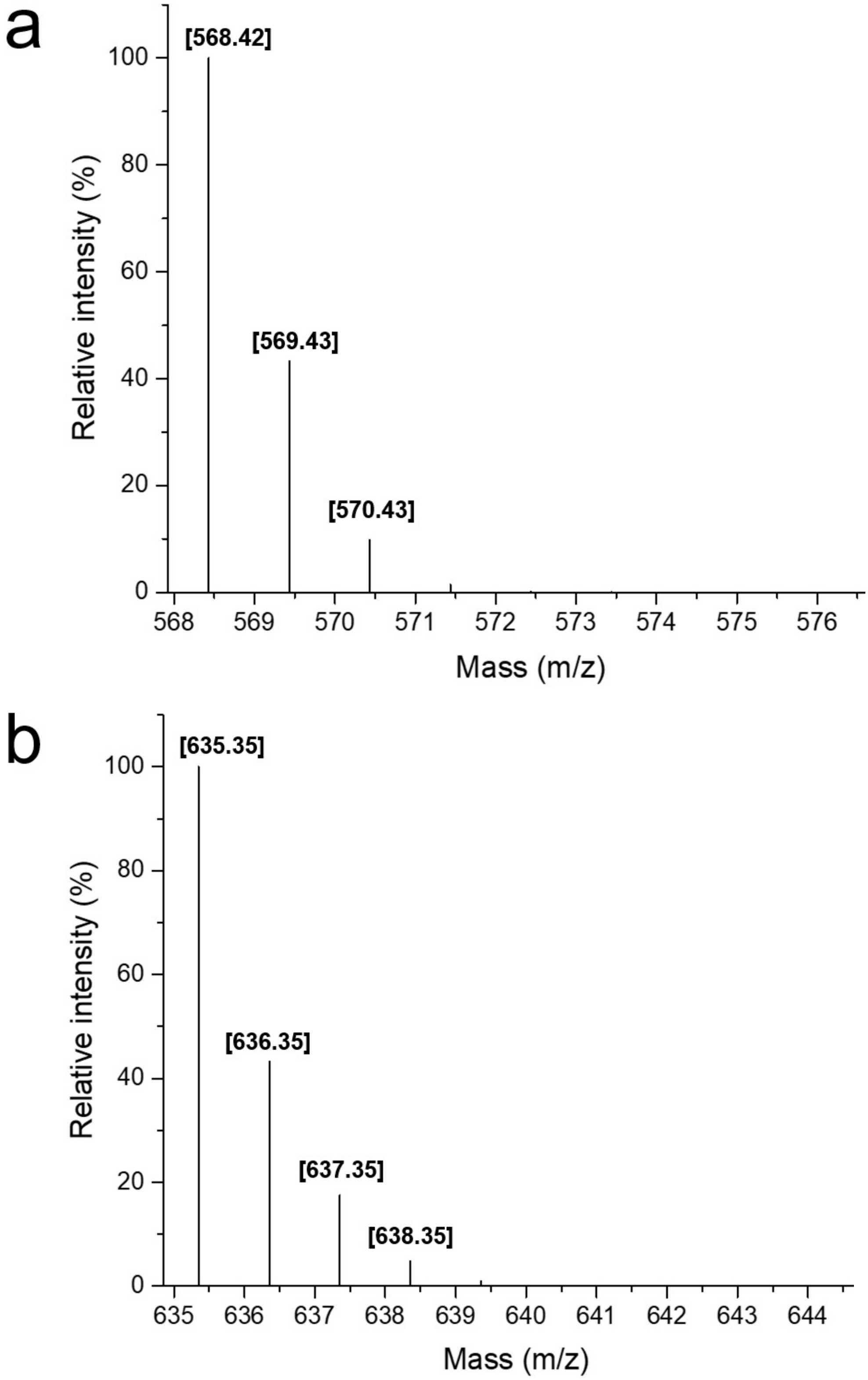

Fig. S9. Isotopic distribution calculated for **a** ($C_{40}H_{56}O_2$, lutein or zeaxanthin) and **b** ($C_{40}H_{52}O_4K^+$, astaxanthin doped with K^+) in "ChemCalc" (http://www.chemcalc.org).

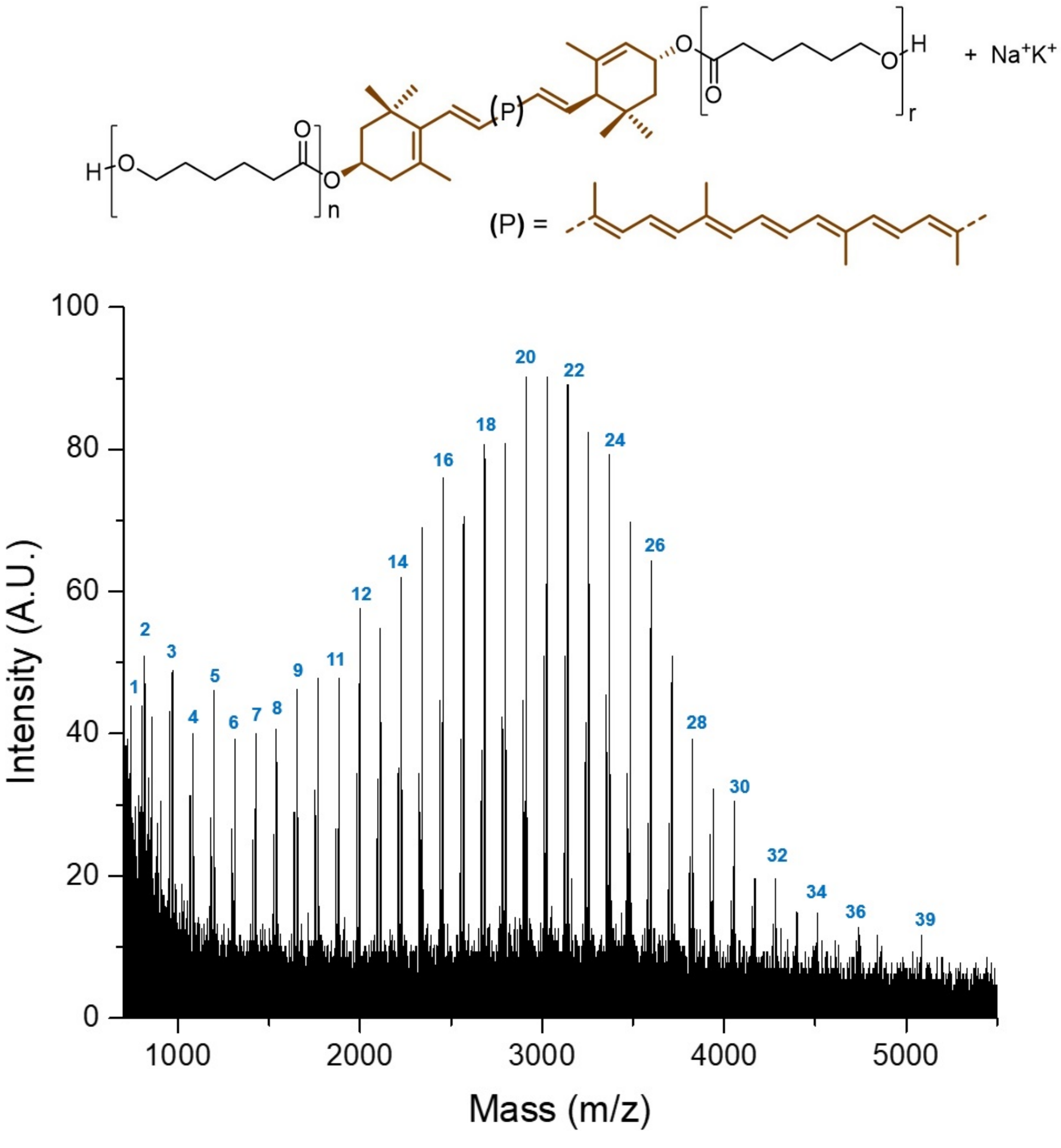

Fig. S10. MALDI-TOF spectrum of lutein–PCL; the number indicates the degree of polymerization (DP) of each peak.

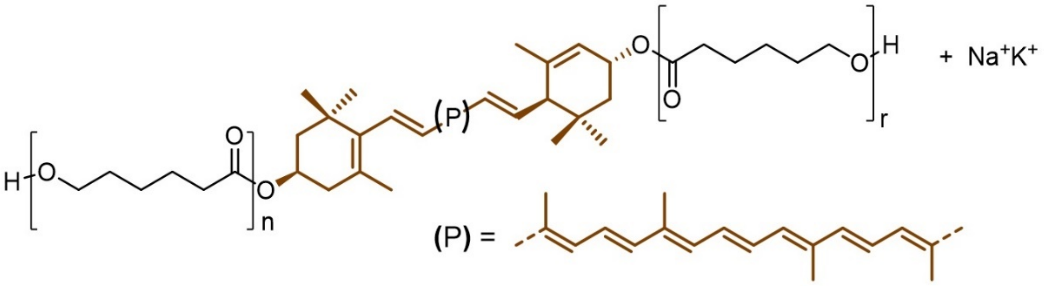

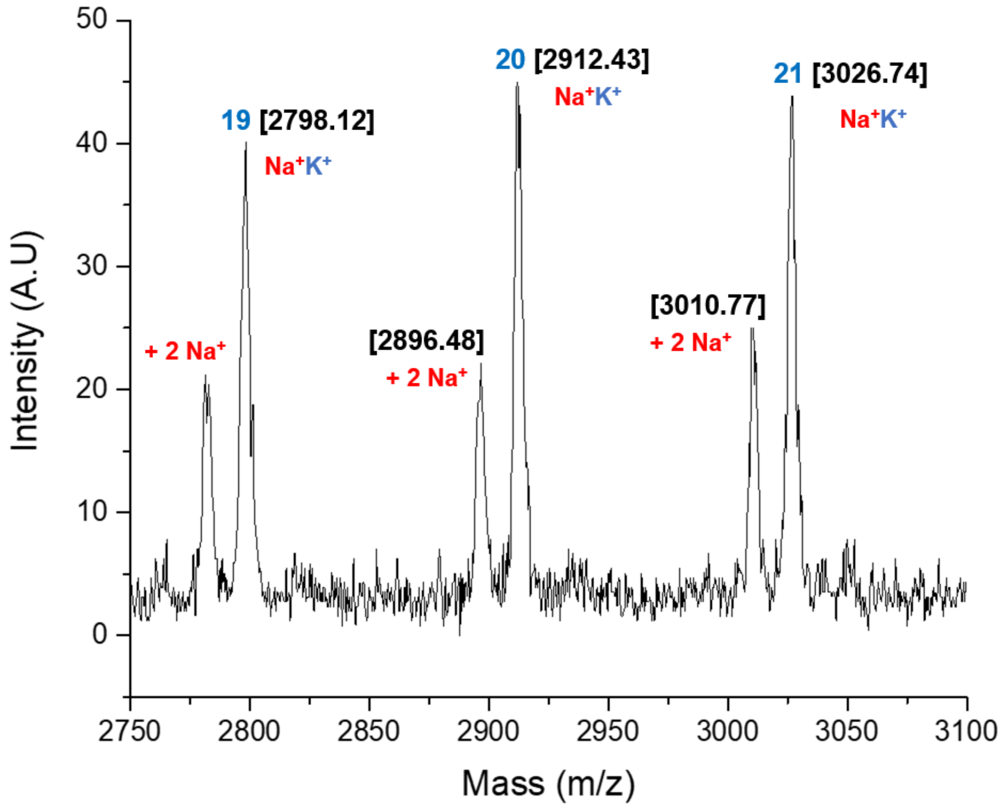

Fig. S11. MALDI-TOF spectrum of lutein–PCL; expanded view for the 2750-3100 m/z fragments of figure S10.

Table S3. MW of the expanded view of MALDI-TOF spectrum of lutein–PCL with DP = 20.

Species	MW (g/mol)	Theoretical MW [a] (2 Na^+)	Experimental (2 Na^+)	Theoretical [a] (Na^+K^+)	Experimental (Na^+K^+)
**DP 19**	2799.62	2781.70	2782.11	2797.67	2798.12
**DP 20**	2913.76	2895.76	2896.48	2911.74	2912.43
**DP 21**	3027.90	3009.83	3010.77	3025.81	3026.74

[a] Calculated by the web service “ChemCalc” (http://www.chemcalc.org).

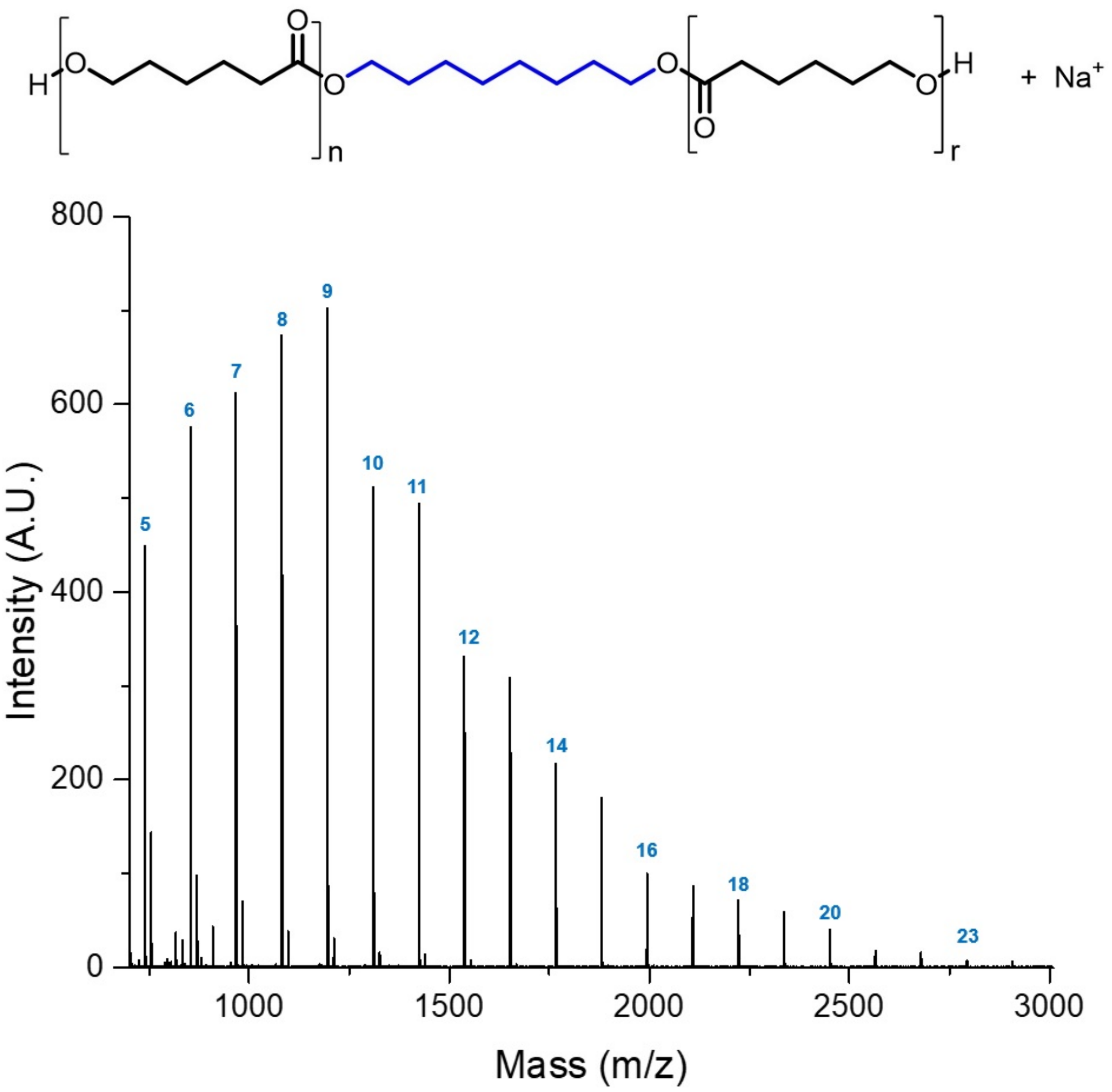

Fig. S12. MALDI-TOF spectrum of 1,8-octanediol-PCL; the number indicates the degree of polymerization of each peak.

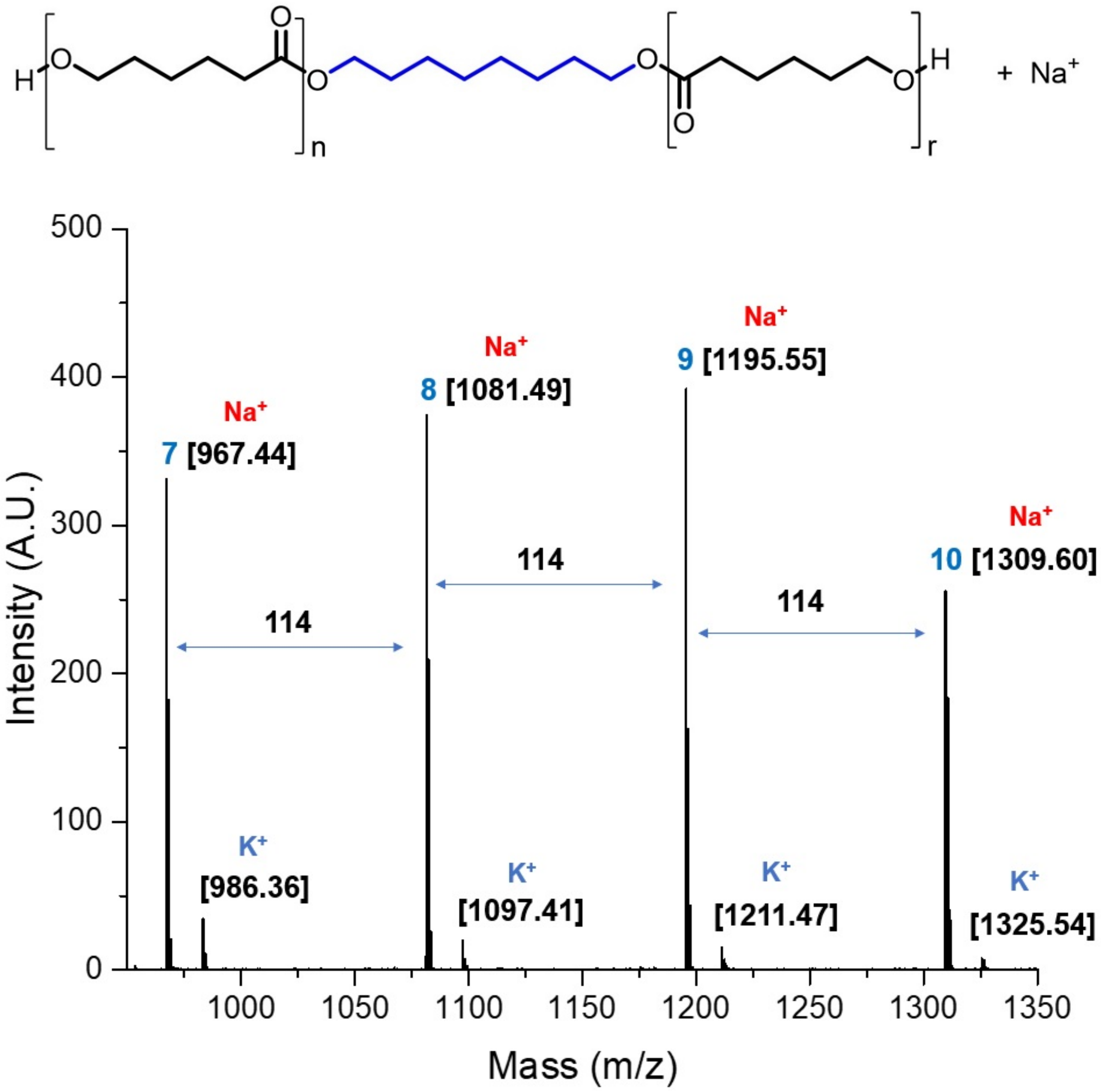

Fig. S13. Expanded view of the 950-1350 m/z fragments of Figure S12 MALDI-TOF spectrum for the 1,8-octanediol-PCL.

Table S4. MW of the expanded view of the MALDI-TOF mass spectrum of the 1,8-octanediol-PCL with DP = 10.

Species	MW (g/mol)	Theoretical MW [a] (Na^+)	Experimental (Na^+)	Theoretical [a] (K^+)	Experimental (K^+)
**DP 7**	945.23	968.22	967.44	984.32	986.36
**DP 8**	1059.37	1082.36	1081.49	1098.47	1097.41
**DP 9**	1173.51	1196.50	1195.55	1212.61	1211.47
**DP 10**	1287.65	1310.64	1309.60	1326.75	1325.54

[a] Calculated by the web service "ChemCalc" (http://www.chemcalc.org).

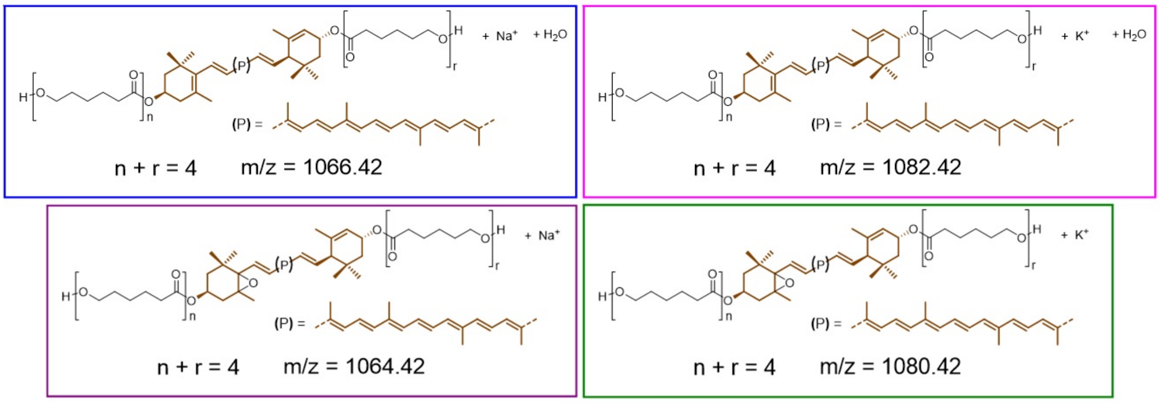

Fig. S14. Tentative structures of “epoxy”-xanthophylls-PCL. The presence of epoxy group contributed with a mass of 16 Da, which did not match with the experimental mass.

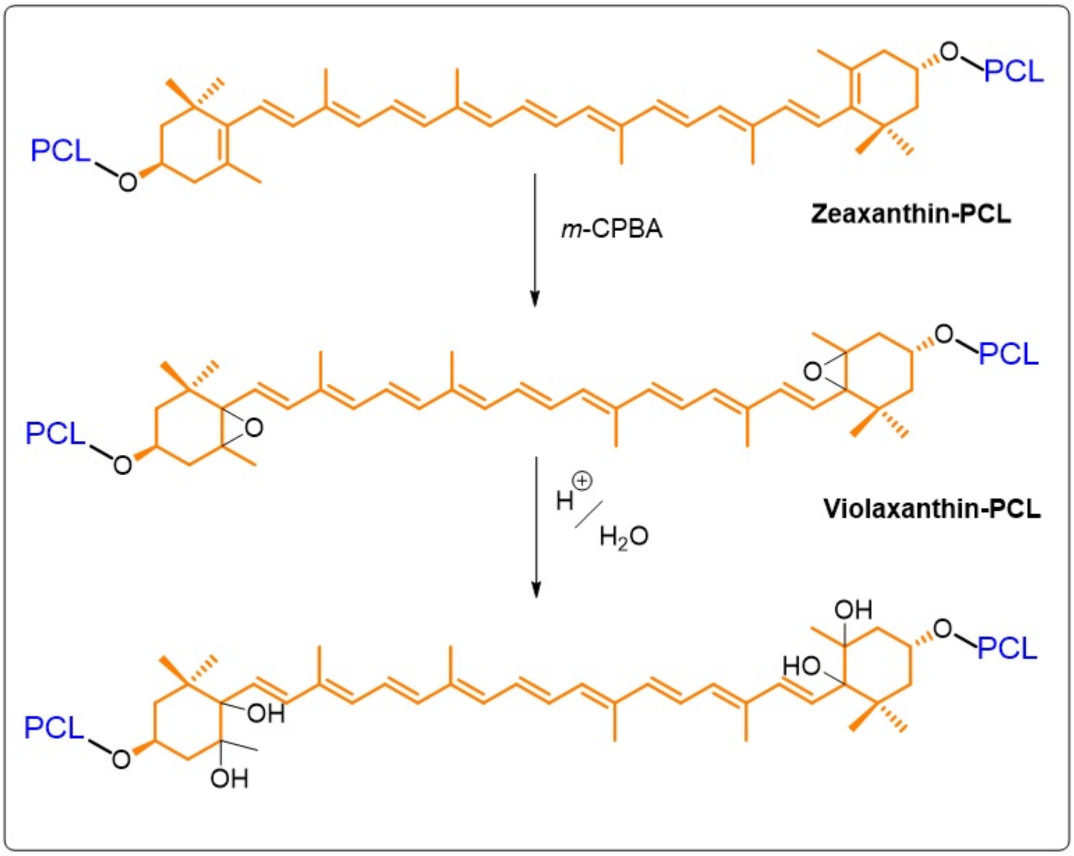

Scheme S1. Functionalization of “epoxy”-xanthophylls-PCL.

Thermal properties of xanthophylls-PCL:

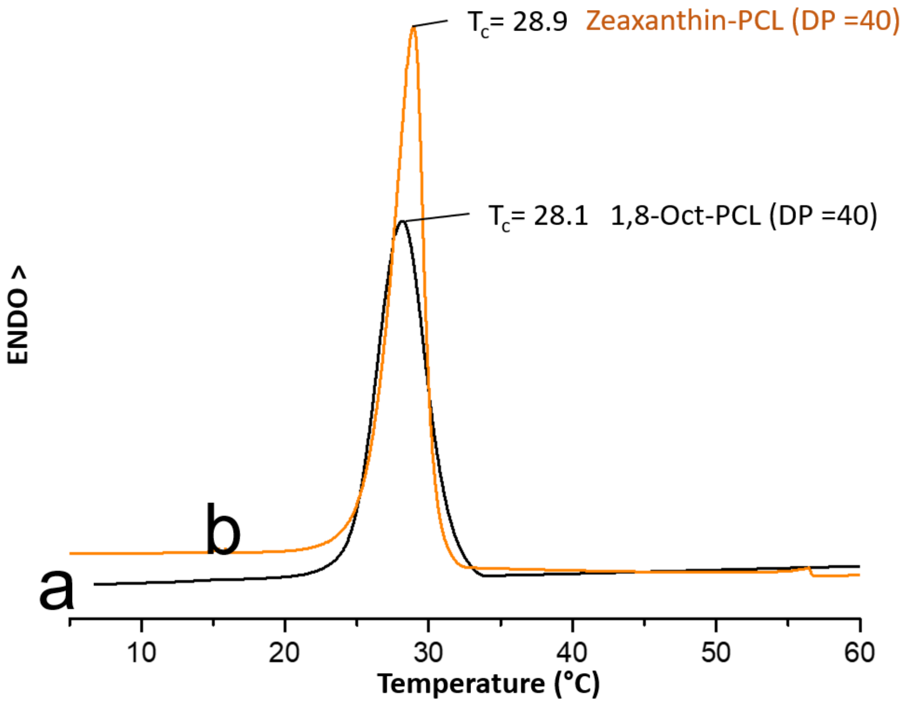

Fig. S15. DSC thermograms (cooling, T_c) of **a**) 1,8-octanediol-PCL (DP = 40), and **b**) zeaxanthin-PCL (DP = 40).

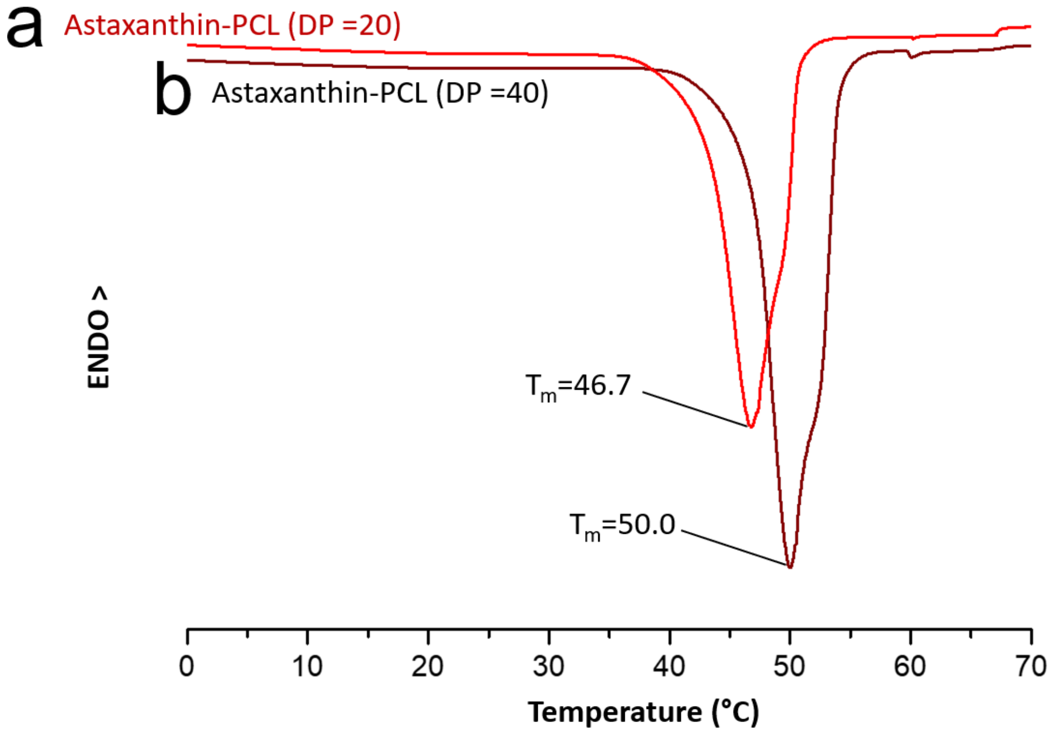

Fig. S16. DSC thermograms of the polyesters derived from astaxanthin.
